# DNA methylation status classifies pleural mesothelioma cells according to their immune profile: implication for precision epigenetic therapy

**DOI:** 10.1186/s13046-025-03310-0

**Published:** 2025-02-18

**Authors:** Maria Fortunata Lofiego, Rossella Tufano, Emma Bello, Laura Solmonese, Francesco Marzani, Francesca Piazzini, Fabrizio Celesti, Francesca Pia Caruso, Teresa Maria Rosaria Noviello, Roberta Mortarini, Andrea Anichini, Michele Ceccarelli, Luana Calabrò, Michele Maio, Sandra Coral, Anna Maria Di Giacomo, Alessia Covre, on behalf of the EPigenetic Immune-oncology Consortium Airc (EPICA) investigators

**Affiliations:** 1 University of Siena, Siena, Italy; 2 BIOGEM Institute of Molecular Biology and Genetics, Ariano Irpino, Italy; 3 Department of Electrical Engineering and Information Technology (DIETI), University of Naples “Federico II”, Naples, Italy; 4 Center for Immuno-Oncology, University Hospital of Siena, Siena, Italy; 5 Sylvester Comprehensive Cancer Center, Miller School of Medicine, University of Miami, Miami, FL USA; 6 Department of Public Health Sciences, Miller School of Medicine, University of Miami, Miami, FL USA; 7 Human Tumors Immunobiology Unit, Department of Experimental Oncology, Fondazione IRCCS Istituto Nazionale Dei Tumori, Milan, Italy; 8 Department of Translational Medicine, University of Ferrara, Ferrara, Italy; 9 Division of Medical Oncology, Department of Medical Oncology, University Hospital of Ferrara, Ferrara, Italy

**Keywords:** Pleural mesothelioma, DNA methylation, Epigenetic, Immunotherapy, Guadecitabine

## Abstract

**Background:**

Co-targeting of immune checkpoint inhibitors (ICI) CTLA-4 and PD-1 has recently become the new first-line standard of care therapy of pleural mesothelioma (PM) patients, with a significant improvement of overall survival (OS) over conventional chemotherapy. The analysis by tumor histotype demonstrated greater efficacy of ICI therapy compared to standard chemotherapy in non-epithelioid (non-E) *vs.* epithelioid (E) PM, although some E PM patients also benefit from ICI treatment. This evidence suggests that molecular tumor features, beyond histotype, could be relevant to improve the efficacy of ICI therapy in PM. Among these, tumor DNA methylation emerges as a promising factor to explore, due to its potential role in driving the immune phenotype of cancer cells. Therefore, we utilized a panel of cultured PM cells of different histotype to provide preclinical evidence supporting the role of the tumor methylation landscape, along with its pharmacologic modulation, to prospectively improve the efficacy of ICI therapy of PM patients.

**Methods:**

The methylome profile (EPIC array) of distinct E (*n* = 5) and non-E (*n* = 9) PM cell lines was analyzed, followed by integrated analysis with their associated transcriptomic profile (Clariom S array), before and after in vitro treatment with the DNA hypomethylating agent (DHA) guadecitabine. The most variable methylated probes were selected to calculate the methylation score (CIMP index) for each cell line at baseline. Genes that were differentially expressed (DE) and differentially methylated (DM) were then selected for gene ontology analysis.

**Results:**

The CIMP index stratified PM cell lines into two distinct classes, CIMP (hyper-methylated; *n* = 7) and LOW (hypo-methylated; *n* = 7), regardless of their E or non-E histotype. Integrated methylome and transcriptome analyses revealed that CIMP PM cells exhibited a substantial number of hyper-methylated, silenced genes, which negatively impacted their immune phenotype compared to LOW PM cells.

Treatment with DHA reverted the methylation-driven immune-compromised profile of CIMP PM cells and enhanced the constitutive immune-favorable profile of LOW PM cells.

**Conclusion:**

The study highlighted the relevance of DNA methylation in shaping the constitutive immune classification of PM cells, independent of their histological subtypes. The identified role of DHA in shifting the phenotype of PM cells towards an immune-favorable state highlights its potential for evaluation in phase I/II clinical trials investigating the efficacy of epigenetic-based ICI combinations to reverse cancer immune resistance mechanisms.

**Supplementary Information:**

The online version contains supplementary material available at 10.1186/s13046-025-03310-0.

## Introduction

Pleural mesothelioma (PM) is an aggressive cancer with a poor prognosis, characterized by a median survival rate of less than a year [1]. Existing treatments, including chemotherapy, radiotherapy, and surgery, have shown limited effectiveness. However, the rising incidence of PM underscores the urgent need for novel and more effective therapies [2]. Immune checkpoint inhibitors (ICI) have shown promise in treating various solid malignancies and are being explored for PM. Initial clinical results demonstrated that ICIs as monotherapies had limited success in improving overall survival (OS) of PM patients [3–5]. However, a dual ICI approach combining the anti-cytotoxic T lymphocyte antigen-4 (CTLA)−4 monoclonal antibody (mAb) ipilimumab and the anti-programmed cell death (PD)−1 mAb nivolumab, tested in the phase III CheckMate 743 trial (NCT02899299), demonstrated greater efficacy, becoming the first-line therapy for unresectable chemo-naïve PM patients in several countries [6]. In fact, combinatorial ICI regimens significantly improved the median OS (mOS) of PM patients, compared to standard chemotherapy (18.1 *vs.* 14.1 months). In particular, an improved survival benefit with combined ICI therapy *vs.* chemotherapy was observed in PM patients with non-epithelioid (non-E) histology (18.1 *vs.* 8.8 months), compared to those with epithelioid (E) histology (18.2 *vs.* 16.7 months) [7]. Despite these promising outcomes, the majority of PM patients failed to derive clinical benefit from ICI or eventually develop resistance to ICI therapy. The different therapeutic effects of ICI might be attributed to an inability of treatments to reverse an “immune-cold” tumor microenvironment (TME), associated in PM with M2-macrophages and myeloid derived suppressor cells (MDSC) infiltration, [8], and lack of infiltrating effector T cells [9]. A favorable influence of the immune contexture in PM clinical outcome is supported by different lines of evidence. First, low abundance of T-helper 2 and high cytotoxic T cells infiltration has been shown to promote improved OS in PM patients [10]. In addition, a high number of B lymphocytes in TME and the presence of Tertiary lymphoid structures (TLS) predicts longer survival in E-PM patients [11]. However, at present, factors determining PM response to immunotherapy remain elusive, but detailed response correlations with genome, transcriptome, methylome and immune landscape features are emerging, including a four-gene inflammatory signature that correlates with survival benefit of PM patients treated with ICI [7]. In this context, a quantitative molecular characterization of PM heterogeneity, by a deconvolution approach, identified this cancer as a mixture of E-PM- and non-E-PM-like cell population, whose proportions are highly associated with the prognosis [12]. That study indicated that the molecular gradients represented by sarcomatoid (S)-score, and epithelioid (E)-score were associated with distinct immune infiltration patterns in PM tumors, with the S-score linked to adaptive immune responses and exhausted T cell infiltration, which may have implications for the potential effectiveness of ICI therapy [12]. In addition, the CpG island methylator phenotype (CIMP) index showed a positive correlation with the S-score, indicating the potential role of epigenetics as key regulator of the heterogeneity of PM tumors and immune microenvironment [8]. Among different epigenetic mechanisms, DNA methylation represents a pivotal epigenetic modification that affects immune cell function and tumor immune evasion. It regulates immune cell differentiation, immune responses, and tumor immune microenvironment (TIME) composition [13]. Targeting DNA methylation in TIME offers various potential avenues for enhancing anti-tumor immunity and reducing immunosuppression [13, 14].

In this scenario, several studies have highlighted that epigenetic drugs, primarily DNA hypomethylating agents (DHA), represent a strategy to increase tumor immunogenicity and reprogram the immune TME [15]. In fact, it has been firmly demonstrated that epigenetic remodeling of cancer cells across various histotypes by DHA, such as decitabine and guadecitabine, induced/up regulated the expression of multiple immune molecules, thereby improving immune recognition of tumor cells [16–19].

Based on this evidence, in this study, we carried out a methylation profiling of human PM cell lines from different histological subtypes to develop an epigenetic classification based on the CIMP index. This approach identified two distinct CIMP (hyper-methylated) and LOW (hypo-methylated) PM classes, independent of their histological subtype. We also found that the transcriptome was associated with the methylome-defined classes, revealing specific biological processes (BP) activated or inhibited in each methylation-based subset. The most notable differences between CIMP *vs.* LOW PM cell lines reflected the predominance of negative regulation of immune activation in the former compared to the latter. In detail, enrichment analysis of genes downregulated by hypermethylation in CIMP *vs.* LOW PM cells identified inhibition of canonical pathways (CP) related to antigen processing and presentation and B cell receptor (BCR) signaling, which could underpin an ICI-resistant phenotype in CIMP PM cells. Interestingly, we found that this immune-compromised, hyper-methylated profile in CIMP PM cell lines could be reverted by DHA treatment, leading to the activation of antigen processing and presentation pathways, as well as inflammatory mediators. Noteworthy, the positive immunomodulatory effect of DHA was also observed in LOW PM cell lines, mainly associated with activation of interferon-mediated signaling and antigen processing and presentation pathways.

Comprehensively, our results provide proof-of-principle evidence supporting a methylation-based classification of PM cells as a novel approach identifying PM tumors potentially more susceptible to ICI therapy. Furthermore, the immunomodulatory activities of DHA treatment could represent a strategy to shift both CIMP and LOW PM cells toward an ideally more ICI-responsive phenotype, providing a rationale to explore a novel epigenetic-based ICI treatment for PM patients, irrespective of their histology.

## Materials and methods

### Tumor cell lines

PM cell lines were established as described [20] from pleural effusions of PM patients treated at the University Hospital of Siena, under approval by the Committee on Human Research (Meso 1, Meso 2, Meso 4, Meso 5, Meso 6, Meso 7, Meso 8, Meso 11, Meso 12, Meso 13) or commercially purchased from American Type Culture Collection Cell Bank (ATCC) (Manassas, VA, USA) (Meso 10 (MSTO-211H), Meso 14 (NCI-H2052), Meso 15 (NCI-H2452) and Meso 16 (NCI-H28)). PM cell lines were categorized into epithelioid (E; 5) and non-epithelioid (non-E; 9) subtypes according to the histology of tumor lesion and based on the expression of epithelial-to-mesenchymal transition (EMT) markers, as previously described [21]. Cells were cultured using HAM’s F-12 medium (Euroclone, Milano), except for Meso 10, Meso 14, Meso 15 and Meso 16 that were grown in RPMI 1640 medium (Thermo Scientific, MA, USA). Both these media were supplemented with 10% heat-inactivated fetal bovine serum (FBS) (Euroclone, Milan, Italy), 2 mM L-glutamine (Thermo Scientific, MA, USA), and 100 µg/µL of penicillin/streptomycin (Euroclone, Milan, Italy). PM cell lines authentication was performed in this study by STR DNA profiling analysis, as described in Additional file 1. Cells were incubated at 37 °C with 5% CO_2_ and passaged at 80–90% confluency. Experiments were conducted using PM cells after the 20th passage.

### In vitro epigenetic drug treatment

Treatment with guadecitabine was performed as previously described [22]. Briefly, PM cell lines, confirmed to be free of mycoplasma contamination (Roche, Indianapolis, USA), were seeded at concentration of 1 × 10^6^/mL in T75 cm^2^ tissue culture flasks (on day 0). Cells were treated with 1 µM guadecitabine (MedChemExpress) every 12 h, twice a day (on days 1 and 2), and were harvested on day 5 for all subsequent analyses. The guadecitabine dose was selected based on our previous studies, in which we demonstrated its demethylating activity and cell viability of at least 70% on a panel of solid tumor cell lines of different histotypes [18, 23]. Control PM cell lines were maintained under the same experimental conditions, without drug administration.

### Clariom™ S human assay for gene-level expression profiling

Microarray profiling was performed on 500 ng of DNAase I-digested RNA using the Affymetrix Clariom™ S human microarray platform (Affymetrix, Santa Clara, CA). Each biotin-labeled sense target was hybridized onto a single GeneChip® Clariom™ S Affymetrix human microarray. After hybridization, the microarrays were washed to remove nonspecifically bound material and were then subjected to image acquisition using the Affymetrix Fluidics Station 450. The acquired images were processed with the Affymetrix GeneChip® Command Console software (provided by Thermo Fisher Scientific, Inc).

### Genome-wide methylation analysis: the Infinium MethylationEPIC array

Genome-wide methylation analysis was performed on 500 ng of DNA isolated from #14 human PM cell lines using the QIAamp DNA Blood Mini Kit (Qiagen, Hilden, Germany). The analysis was conducted with the Infinium Human Methylation EPIC 850 k (EPIC) array, following the manufacturer’s standard protocols. In detail, EPIC 850 k array is based on Illumina’s BeadChip technology, which employs a dual-probe design to capture the full spectrum of DNA methylation at the single-CpG-site level with high resolution. The Infinium design uses specific probes to interrogate each CpG site, and the signal intensity emitted by their interaction is then measured to generate beta values (β), defined as β = M/(M + U + 100), where M represents the intensity corresponding to methylated sites and U represents unmethylated sites, indicating the relative degree of methylation at a given locus. β values range from 0 to 1, representing fully unmethylated and fully methylated states, respectively. The β values were normalized using the functional normalization approach [24]. Subsequently, the β values of cell lines (at baseline) were used to estimate the most variable probes, and a methylation score was calculated by summing the methylation levels of the selected probes. Raw intensities were processed using the *minfi* R package (v 1.42.0) [25]. The methylation data were normalized by using the functional normalization approach implemented in the *preprocessFunnorm()* function of the R package *minfi* [25]. Probes of low quality, those located in the X and Y chromosomes, and probes known to have single nucleotide polymorphisms (SNPs) at the CpG site or that are cross-reactive, were filtered out.

### Data analysis

#### Classification of PM cell lines based on the CIMP index

Normalized and filtered probes were used to estimate the most variable ones, based on the difference between the 10th and 90th percentiles of the data. Approximately 1% of the total probes, identified as the most variable, were selected to calculate the methylation score (CIMP-index) for each cell line at baseline by summing the β values across these selected probes. PM cell lines were then categorized into two groups according to the median CIMP index: those with a methylation score above the median (CIMP) and those with a methylation score below the median (LOW).

### Differential methylation analysis

Differential methylation analysis was performed to identify DM probes between CIMP *vs.* LOW untreated PM cell lines, as well as between DHA-treated and untreated PM cell lines within both CIMP and LOW groups, using the R package *limma* [26] by fitting a linear model to the matrix of M values. Probes with a *p*-value < 0.05 and an absolute log2 fold change |log2FC|> = 1 were selected as DM. Gene ontology (GO) enrichment analysis was performed using the *gometh()* function from the R package *missMethyl* [27], focusing on DM probes overlapping the promoter region (5’UTR, TSS1500, TSS200). BP with a *p*-value < 0.01 were then selected.

### Differential expression analysis

Differential expression analysis was performed to identify differentially expressed genes (DEG) between CIMP *vs.* LOW untreated PM cell lines, as well as between DHA-treated and untreated PM cell lines within both CIMP and LOW groups, using Transcriptome Analysis Console (TAC) Software (Applied Biosystems, Thermo Fisher Scientific). Genes with a *p*-value < 0.05 and |log2FC|> = 1 were selected as DE. Statistical analysis was performed by Mann–Whitney test.

### Integrative analysis of methylation and expression data

Results from the methylation and expression analyses for each comparison were integrated by selecting genes that were both DE and DM in the promoter region. Starburst plots displayed DM probes overlapping the promoter region. The -log10(*p*-value) of the upregulated genes and the log10(*p*-value) of downregulated genes were plotted against the -log10(*p*-value) of the hyper-methylated probes and the log10(*p*-values) of down-methylated probes. Functional analysis of these expression/methylation-concordant gene modules was conducted using QIAGEN Ingenuity Pathway Analysis tool (IPA) [28].

### GO terms analysis

IPA Core analysis was performed to identify, at transcriptomic level, upstream regulators (UR) predicted to be activated (Z-score ≥ 2) or inhibited (Z-score ≤ − 2) at the constitutive level in CIMP *vs.* LOW PM cell lines, as well as within both CIMP and LOW classes before and after DHA treatment. GO term enrichment, focusing on BP, was conducted utilizing the EnrichR web-tool [29]. Significative BP terms (*p*-value < 0.05) were ranked based on their combined score value, calculated by multiplying the log of the *p*-value (computed with the Fisher exact test) by the Z-score from our test correction. The top 50 BP terms were then further analyzed. Moreover, IPA Core analysis was performed on hyper-methylated down-regulated genes, detected through integrative analysis to identify CP inhibited (Z-score ≤ − 2) at the constitutive level in CIMP *vs.* LOW PM cell lines and, on hypomethylated and upregulated genes to define CP activated (Z-score ≥ 2) in CIMP and LOW PM cell lines after DHA treatment.

### Analysis in PM lesions from the TCGA-MESO cohort

Illumina 450 K methylation array data of The Cancer Genome Atlas (TCGA) MESO cohort (*n* = 87 samples) were downloaded using the TCGABiolinks R package [30]. Probes located on the X and Y chromosomes, in open sea regions, containing SNPs at the CpG site, identified as cross-reactive, or bowtie2 multi-mapped were removed. The remaining filtered probes were used to estimate the most variable ones based on the difference between the 10th percentile and the 90th percentile of the data. A total of #2,285 highly variable sites were selected to calculate a methylation score for each sample by summing the β values across these variable sites. Samples with a methylation score higher than the median value were classified as hyper-methylated (CIMP, *n* = 44), while the ones with a lower value were classified as hypo-methylated (LOW, *n* = 43).

Expression data of the TCGA-MESO cohort were downloaded from cBioPortal (http://www.cbioportal.org, accessed on August 1, 2024). The file ‘data_mrna_seq_v2_rsem.txt’ was used to explore the differences in the expression profiles between CIMP and LOW samples and to estimate the abundance of eight immune cell populations and two stromal cell populations in the tissues. Differential expression analysis was performed using the Wilcoxon test to compare the CIMP samples with the LOW ones. Then, the genes sorted according to the Wilcoxon statistics were used for a gene set enrichment analysis with the clusterProfiler R package [31]. The immune and stromal subpopulation abundances in the tissue of each sample were estimated using the MCPcounter R package deconvolution analysis [32]. The same analysis was also performed by considering the top 25% (*n* = 22) and the bottom 25% (*n* = 22) methylated tumor samples. The samples were ranked according to their methylation score. The Log-rank (Mantel-Cox) test was performed to assess if there was a significant difference between the groups. mOS rates were estimated by Kaplan–Meier analysis, with two-sided 95% confidence intervals (CI) calculated using a normal approximation method; survival curves were compared using the log rank test. *p* < 0.05 were considered statistically significant.

### Gene expression analysis by quantitative Real-Time RT-PCR

Relative quantification by Real-Time PCR was performed using 21.8 ng of retrotranscribed RNA in a final reaction volume of 20 μL with TaqMan Fast Advanced Master Mix (Applied Biosystems, CA, United States). Relative gene expression levels were quantified by the 2^-ΔΔCT method, where ΔCT represents the difference between the CT values of the target gene and the reference gene β-actin; ΔΔCT reflects the difference in ΔCT between treated *vs.* untreated cells (FC). Gene expression was considered upregulated (FC ≥ 1.5) or downregulated (FC ≤ 0.5) if it exhibited a 1.5-fold increase or decrease, respectively. All relative quantification analyses were performed using the QuantStudio ™ 5 Real-Time RT-PCR System (Applied Biosystems ™, CA, USA) and its analysis software. Gene-specific probes for human endogenous retroviruses (HERV) and interferon-stimulated genes (ISG) were selected from the Taqman Gene Expression Assay database (Applied Biosystem, Foster City, CA, USA) (Table 1) or designed using Custom Assay Design (Applied Biosystem, Foster City, CA, USA) (Table 2).

**Table 1:** Gene specific assays for ISGs and HERVs expression

**ISG gene name**	**Assay ID**
DDX58	(ID Hs01061436_m1)
IFIT1	(ID Hs03027069_s1)
IFIT2	(ID Hs01922738_s1)
IFI27	(ID Hs01086373_g1)
IFI6	(ID Hs00242571_m1)
OAS2	(ID Hs00942643_m1)
IRF7	(ID Hs01014809_g1)
IFITM1	(ID Hs00705137_s1)
ISG15	(ID Hs01921425_s1)
ISG20	(ID Hs00158122_m1)
IFI44	(ID Hs00951349_m1)
**HERV gene name**	**Assay ID**
Syncytin-1	Hs01926764_u1
Syncytin-2	Hs01942443_s1
ENV-V2	Hs04935552_m1
ERV9-1	Hs03921574_s1
ENV-MER34-1	Hs01029273_s1
ERV-Fb1	Hs05577546_g1

**Table 2:** Customized gene specific assay for HERV-FXA34 expression

Accession Number	Assay ID	Target Sequence
ERV-FXA34 (U29659.1)	APRWNMA	CTCCATTAGTAGCAGTTCCTCTCCCTACCCCCTTTAATTATACTATAAATTCATCAACCCCTATACCACCGGTCCCAAAAGGACAGGTCCCACTATTCTCAGACCCTATAAGACATAAGTTCCCATTCTGTTACTCTACCCCAAATGCCTCTTGGTGTAACCAGACTAGGATGCTTACCAGCACCCCGGCACCGCCCAGGGCTACTTCTGGTGTAACTCCACGCTAACTAAAGTTCTTAACTCAACTGGTAATCACACCTTGTGCTTACCCATCTCTCTCATCCCTGGCCTGACCCTATATAGTCAGGATGAACTTAGCCATCTGCTAGCCTGGACCGAGCCAAGGCCACAAAATAAAAGCAAATGGGCTATTTT

### Western blot analysis

Total proteins were extracted from 4 selected PM cell lines using the RIPA Lysis and Extraction Buffer Protocol (Thermo Scientific, MA, USA). Total protein lysates were quantified with the Pierce™ Rapid Gold BCA Protein Assay Kit (Thermo Scientific, MA, USA), according to the manufacturer’s guidelines, by the iMark™ Microplate Reader (Bio-Rad, Hercules, CA, USA). Total protein load (20 ug) was detected using the stain-free blot application of the ChemiDoc Imaging System (Bio-Rad, CA, USA) and utilized for target protein normalization. For immunoblotting, membranes were incubated 1 h at room temperature with the following primary antibodies, anti-human ISG15 rabbit (1:1000) (PA5-31,865, Invitrogen, Waltham, MA, USA. Detection of the chemiluminescent signal was performed using a goat anti-rabbit IgG (H + L)-HRP Conjugate secondary antibody (1:3000) (#170–6516, Bio-Rad, CA, USA) and the Clarity™ Western ECL Substrate (Bio-Rad, CA, USA), with visualization on the ChemiDoc Imaging System.

### Soluble factors analysis

Soluble immune modulators in supernatants of untreated and DHA-treated CIMP and LOW PM cells were measured by immunoassays. In detail, interferon γ-induced protein (IP)−10 was quantified using the automated ELLA platform (ProteinSimple, Bio-Techne, Minneapolis, MN, USA) according to the manufacturer’s protocol and IFN-γ, interleukin (IL)−2 and IL-6 were measured with the Human ProQuantum Immunoassay Kit (Invitrogen, Waltham, MA, USA) following the manufacturer’s instructions.

## Results

### Methylation-based classification of PM cell lines

**Fig. 1:**
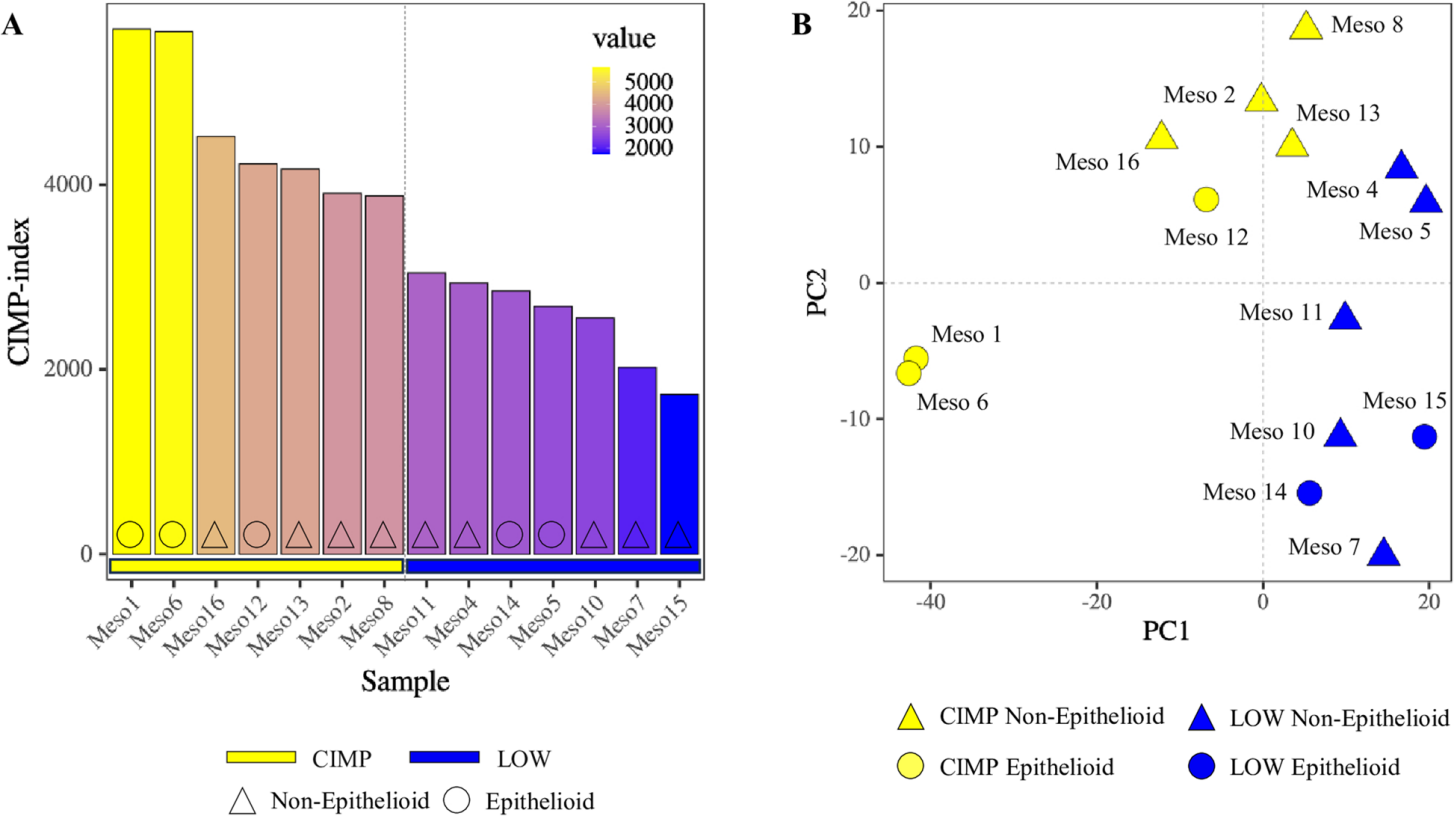
Analysis of methylation patterns (CIMP-index) in PM cell lines. Starting from EPIC array data, the interleaved bar plot represented the CIMP-index, for each cell line, calculated by summing the β values of the most variable methylated probes, that represented approximately 1% of the total probes. The β values were normalized by using the functional normalization approach. The interleaved bar plots of PM cell lines are displayed in descending order of the methylation index, from the highest to the lowest value, and classified in CIMP (yellow) and LOW (blue) groups according to their methylation level, separated by the dotted line. Each bar represents a single epithelioid (circle) or non-epithelioid (triangle) PM cell line at the constitutive level (**A**). Dimensionality reduction was performed applying PCA on the most variable methylated probes among all PM cell lines. Each symbol on the graph represented a cell line categorized by its methylation status: CIMP (yellow) or LOW (blue), and by its histopathological variant: epithelioid (circle) or non-epithelioid (triangle) (**B**)

The methylome analysis identified #7,850 probes as the most variably methylated among all investigated PM cell lines, that were used to calculate the CIMP-index of each cell line. PM cell lines were then stratified into two classes: hyper-methylated (CIMP; *n* = 7) and hypo-methylated (LOW; *n* = 7) based on their CIMP-index higher or lower than median value, respectively (Fig. 1 A). Principal component analysis (PCA) demonstrated that this methylation-based PM cell lines stratification was independent from their histopathological categorization, indeed E and non-E PM cell lines were homogeneously distributed among above median and below median CIMP index values (Fig. 1 B). Among the mapped methylated probes, #63,154 were significantly (*p*-value < 0.05) DM (*n* = 58,710 hyper- and *n* = 4,444 hypo-methylated) in CIMP *vs.* LOW PM cell lines confirming the stratification of these cell lines into PCA-defined groups, independent of their histological classification into E and non-E subtypes (Additional file 2).

**Fig. 2:**
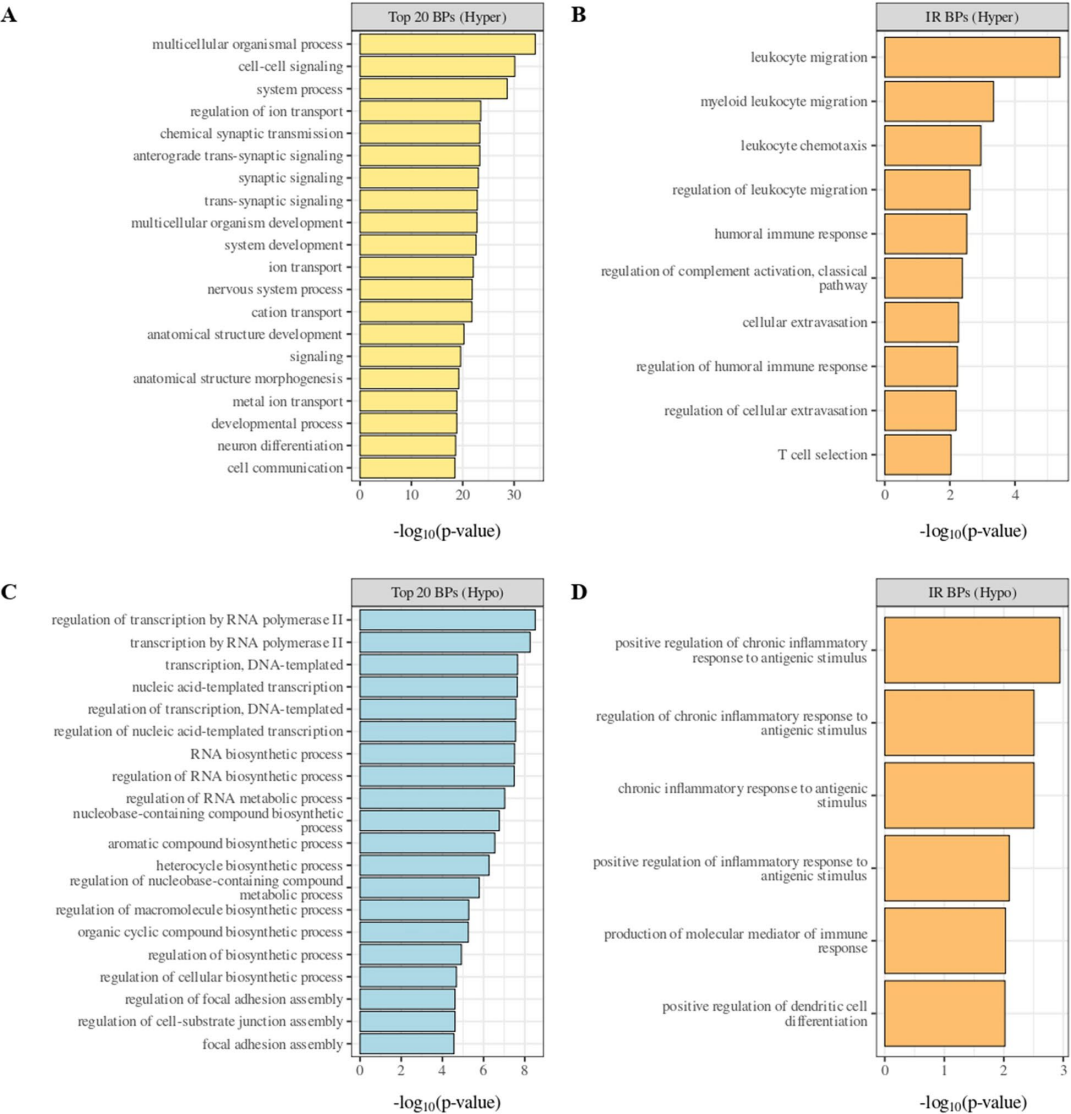
Enrichment analysis of hyper-methylated (**A**, **B**) and hypo-methylated (**C**, **D**) probes in the promoter region of CIMP *vs.* LOW PM cell lines. The top 20 biological processes (BP) enriched by hyper- (**A)** or hypo- (**C**) methylated probes in CIMP *vs.* LOW PM cell lines were identified. The top 10 immune-related (IR)-BPs enriched by hyper-methylated probes (**B**) and the top 6 immune-related (IR)-BP enriched by hypo-methylated (**D**) probes in CIMP *vs.* LOW PM cell lines were identified

To explore the biological effects of the identified DM probes, we examined their distribution across the genome based on their relation to CpG islands (Additional file 3) and gene functional regions (Additional file 3) in CIMP *vs.* LOW PM cell lines. Results showed that the hyper-methylated probes in the CIMP PM cell lines, compared to the LOW ones, were enriched in the 1st Exon (*p*-value < 2.2e-16, proportion test), 5’ UTR (*p*-value < 2.2e-16, proportion test), TSS200 (*p*-value < 2.2e-16, proportion test) and TSS500 (*p*-value < 2.2e-16, proportion test) compared to the hypo-methylated ones. In addition, the hyper-methylated probes were primarily located in CpG island regions (*p*-value < 2.2e-16, proportion test) and north shore regions (*p*-value = 7.474e-08, proportion test). To comprehensively investigate the methylation-driven biological differences of CIMP *vs.* LOW PM cell lines, an enrichment analysis was conducted on DM probes located in the promoter region. Results highlighted multiple processes involved in cell–cell interaction signaling, cell differentiation and chemical synaptic transmission signaling, impacted by hyper-methylated genes, or on biosynthetic and transcription process impacted by hypo-methylated genes, in CIMP *vs.* LOW PM cell lines (Fig. 2 A, C). Focusing on the immune-related (IR) processes category, comparing CIMP *vs.* LOW PM cell lines, the enriched processes impacted by hyper- or hypo-methylated probes were mainly involved in the regulation of humoral immune response, T cell selection and leukocyte migration/chemiotaxis or inflammatory response to antigenic stimulus, dendritic cell (DC) differentiation and production of molecular mediator of immune response, respectively (Fig. 2 B, D).

### Transcriptomic profiles of CIMP vs. LOW PM cell lines

**Fig. 3:**
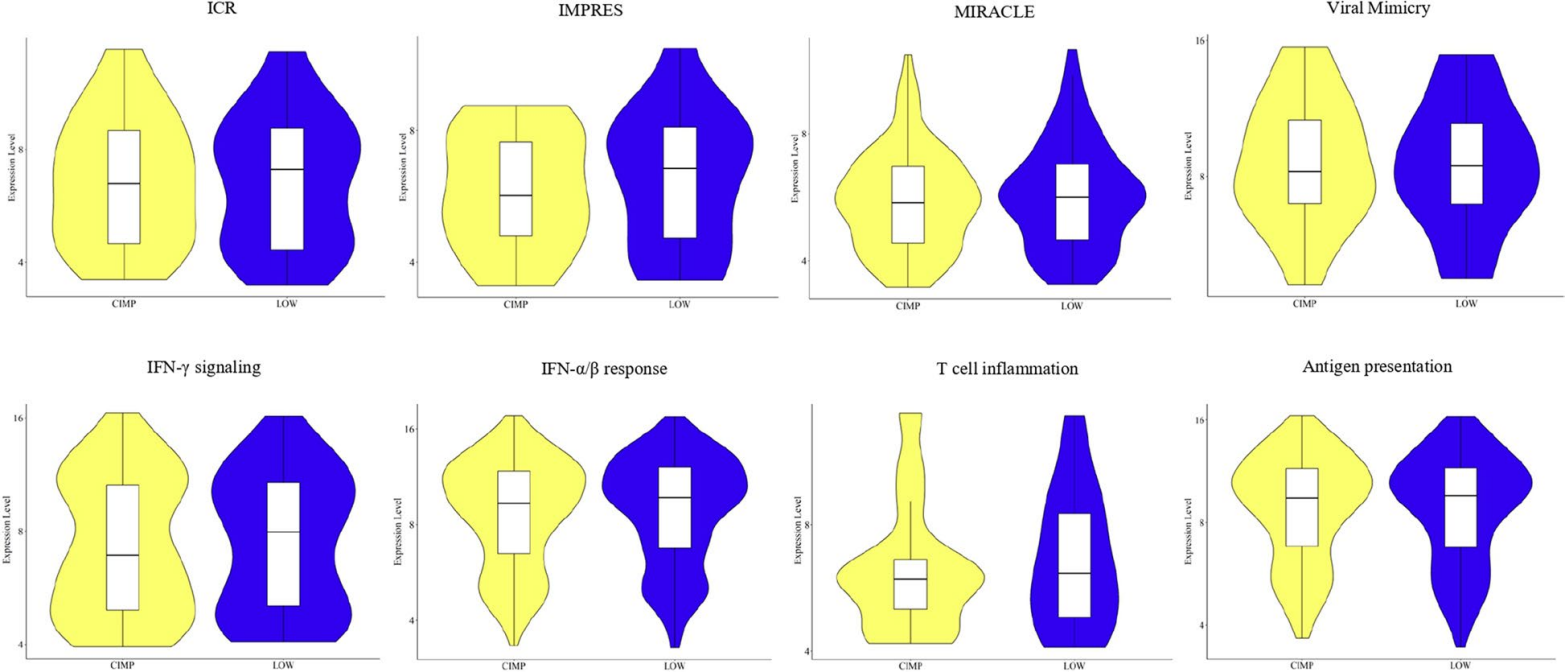
Analysis of predictive ICI response and of tumor immunogenicity gene signatures in CIMP and LOW PM cell lines. Violin plots showed the expression of genes involved in the ICR [33], IMPRES [34], MIRACLE [35], viral mimicry [36], IFN-γ signaling [37], IFN-α/β response [38], T-cell inflammation [39] and antigen presentation [38] signatures in CIMP (yellow) and LOW (blue) PM cell lines

The functional impact of the different immune profiles of CIMP and LOW PM cell lines, shaped by DNA methylation, was confirmed by the observed differential expression of selected signatures associated with tumor immunogenicity or predictive of ICI response. Indeed, the ICR [33], IMPRES [34], MIRACLE [35], viral mimicry [36], IFN-γ signaling [37], IFN-α/β response [38], T-cell inflammation [39], and antigen presentation [38] were enriched in LOW PM cell lines compared to CIMP cell lines (Fig. 3 A). As protein validation of these results was the expression of ISG15 exclusively observed in investigated LOW (*n* = 2) compared to CIMP (*n* = 2) PM cell lines, by Western Blot (Additional file 4).

**Fig. 4:**
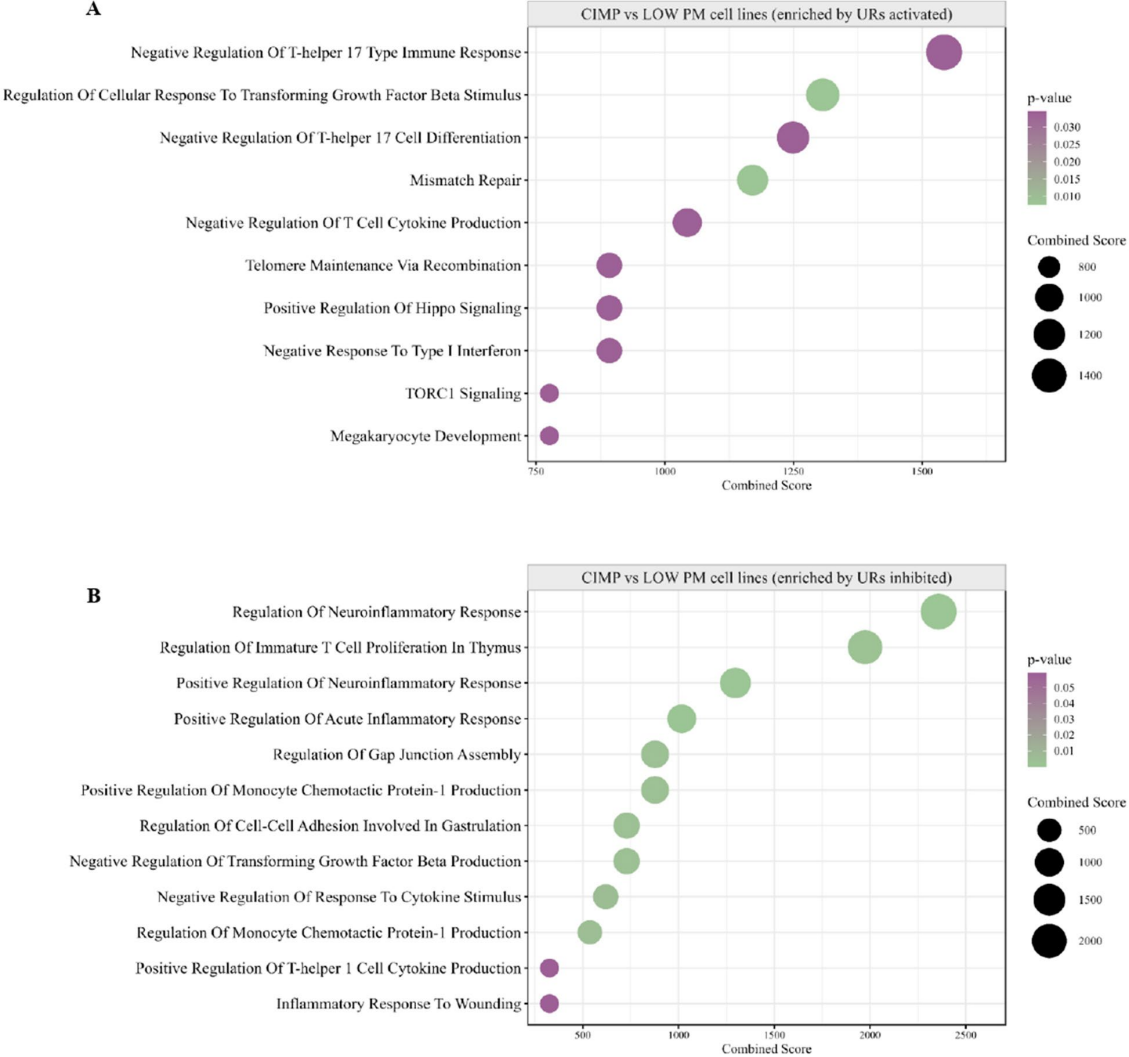
Representative functional categories enriched by differentially activated or inhibited UR in CIMP *vs.* LOW PM cell lines. Dot plot of representative GO terms enriched by activated (**A**) or inhibited (**B**) upstream regulators (UR) in CIMP *vs.* LOW PM cell lines. The color scale from purple to green indicated the ranges of significant *p*-value (*p* < 0.05) and the size of each single dot correlated with the combined score

Moreover, to comprehensively examine the phenotypic differences of DM PM cell lines, a comparative gene expression analysis of CIMP *vs.* LOW PM cell lines was performed, identifying #649 up-regulated genes and #595 down-regulated genes. Bioinformatic IPA core analysis was carried out to predict the constitutive functional status (activation or inhibition) of UR in CIMP *vs.* LOW PM cells lines and identify their associated BP (Fig. 4 A). Results showed that in CIMP PM cells, activated (Z-score ≥ 2) UR were mainly associated with the BP involved in shutting down of the immune response (i.e., negative response to type I interferon, negative regulation of T-helper (Th)−17 type immune response, and negative regulation of T cell cytokine production), in cancer cell invasion and drug resistance (i.e., Hippo signaling pathway), and in tumor progression (i.e., TORC1 signaling) (Fig. 4 A; Additional file 5). Likewise, UR inhibited (Z-score ≤ − 2) in CIMP PM cell lines enriched BP linked to the acute inflammatory response, suppression of Th-1 cells and secretion of pro-inflammatory cytokines (i.e., IL-6, Th-1 cytokine, monocyte chemotactic protein-1) (Fig. 4 B; Additional file 5). Similarly, in CIMP *vs.* LOW PM cell lines, several CP were inhibited, including IL-17 signaling, PD-1/PD-L1 cancer immunotherapy pathway, T Cell Receptor (TCR) and BCR signaling, that exert a pivotal role in coordinating an efficient adaptive cell-mediated immune response, and non-canonical NF-κB signaling, implicated in T cell development. Additionally, the mitotic G2/M phases CP were also inhibited (Additional file 6). Among the predicted activated CP, in CIMP *vs.* LOW PM cell lines, were those related to RORA, RAS processing and PPAR signaling, primarily involved in tumor cell proliferation and invasion [40, 41]. Notably, oncogenic NOTCH1 signaling and cellular stress response pathways (i.e., cytoprotection by HMOX1, and p75 NTR receptor-mediated signaling) were activated in CIMP compared to LOW PM cell lines (Additional file 6).

### Tumor methylation profiles predict survival and influence immune profiles in the TCGA-MESO cohort

**Fig. 5:**
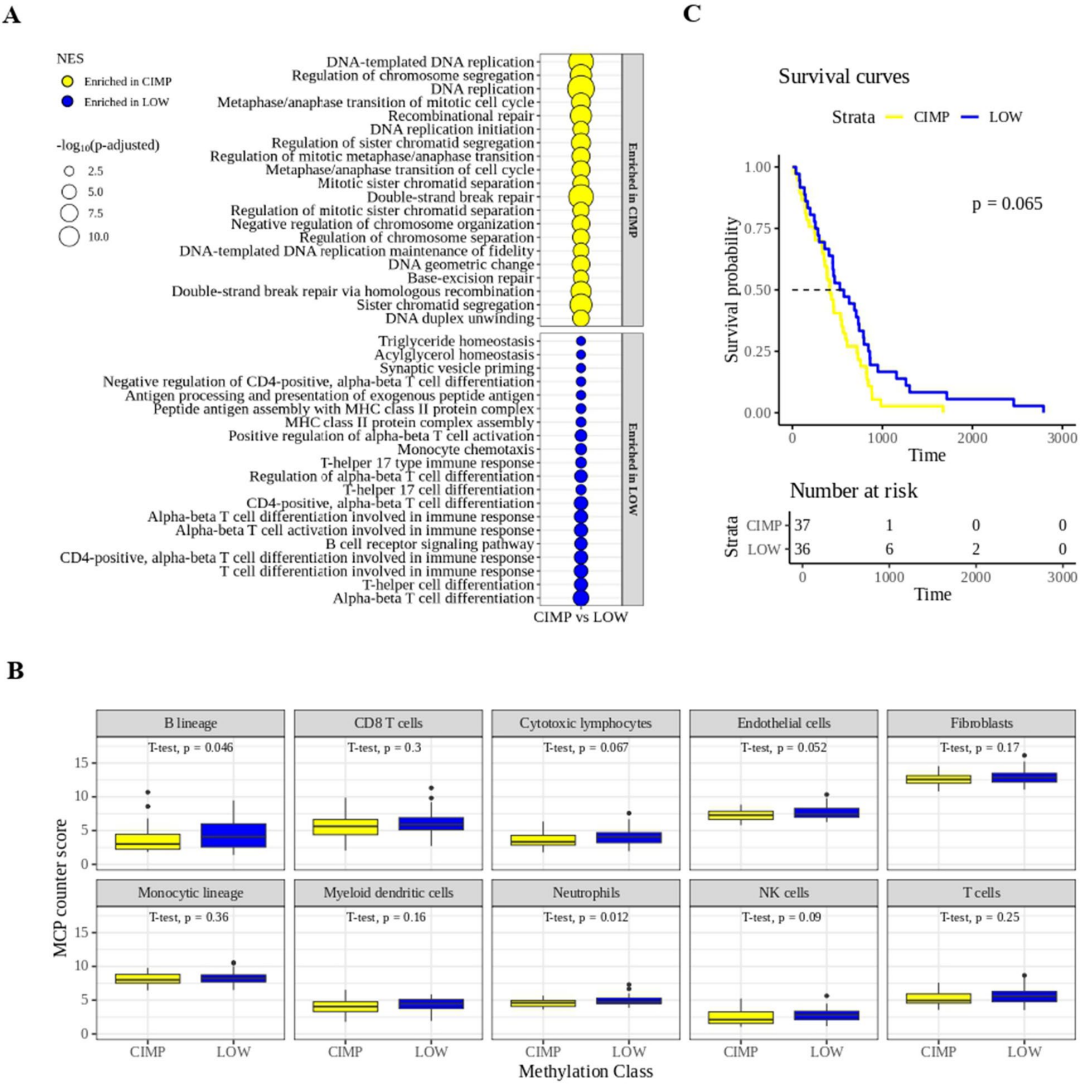
Association between tumor methylation and immune phenotypic profiles and survival in the TCGA-MESO cohort. Gene set enrichment analysis of the differentially expressed (DE) genes in the hyper-methylated (CIMP) and the hypo-methylated (LOW) tumor samples from PM patients of the TCGA-MESO cohort. The top 20 biological processes (BP) enriched in CIMP (yellow) or in LOW (blue) were shown (**A**). Tumor microenvironment deconvolution of immune and stromal cell fractions in CIMP (yellow) and LOW (blue) PM tissues from the TCGA-MESO cohort’ patients (**B**). Kaplan–Meier curve of the OS of CIMP (yellow; #37) and LOW (blue; #36) TCGA patient’s cohort (**C**). Statistical analysis was performed by the Log-rank (Mantel-Cox) test

To investigate the role of the LOW and CIMP methylator phenotypes and of their related immune profile, identified in PM cell lines, in contributing to shape the methylation phenotype and immune contexture of PM tumors, multi-omics profiling of #87 tumor lesions from PM patients in the TCGA-MESO cohort was exploited. Patients were stratified based on their tumor methylation score and classified, according to the median methylation value, in hyper-methylated (CIMP) and hypo-methylated (LOW) classes. This classification was independent of the PM histological subtypes. Consistent with the transcriptional analyses performed on CIMP *vs.* LOW PM cell lines (Fig. 4), we observed an immune-low transcriptional profile in CIMP *vs.* LOW tumor lesions from PM patients, characterized by the enrichment of pathways preferentially involved in regulating processes such as cell cycle and DNA repair mechanisms (Fig. 5 A, upper panel). At the same time, we observed an immune-high transcriptional profile in LOW *vs.* CIMP tumor lesions of PM patients, characterized by the enrichment of several immune pathways regulating T cell differentiation, antigen processing and presentation mechanisms, and B cell signaling (Fig. 4 A, bottom panel). In line with these findings, TME deconvolution analysis of data from the TCGA-MESO cohort revealed that PM lesions in the LOW class exhibited a higher density of selected immune cell infiltrate compared to CIMP, with significant (*p* < 0.05) differences observed for B cells and neutrophils (Fig. 5 B). These differences become significant (*p* < 0.05) also for cytotoxic lymphocytes and myeloid dendritic cells when we considered the top 25% hyper-methylated CIMP *vs.* top 25% hypo-methylated LOW PM lesions (Additional file 7). Noteworthy, these methylation-associated differences of immune aspect of CIMP *vs*. LOW PM lesions seem to affect clinical outcomes in the TCGA-MESO cohort, with a shorter mOS (459 days; 95% confidence interval (CI): 0.95–2.49) for CIMP patients [42], compared to a longer mOS (689 days; 95% CI: 0.42–1.05) for LOW patients (*p* = 0.065) (Fig. 5 C).

### Integrative analysis of promoter methylation level and transcriptomic profiles in CIMP vs. LOW PM cell lines

**Fig. 6:**
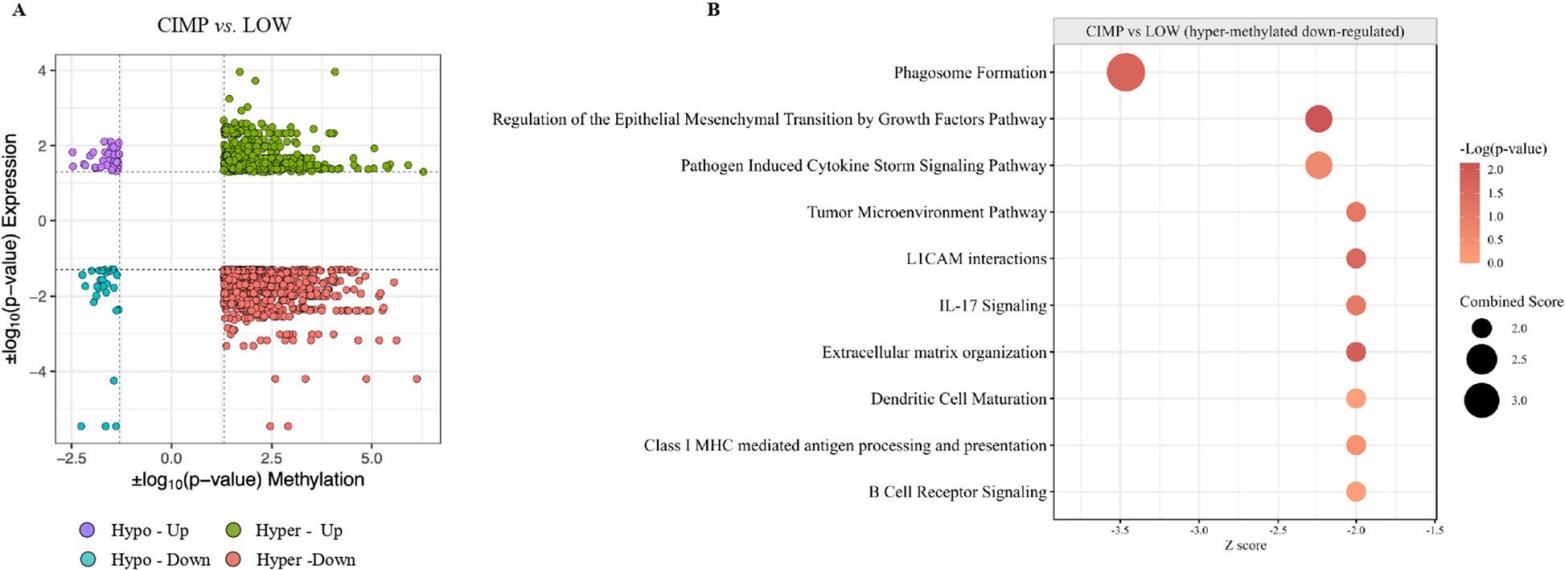
Integrative methylation and expression analysis. The starburst plot showed results of the integrative analysis of promoter methylation and transcriptomic data in CIMP *vs.* LOW PM cell lines. x and y axes represented the |log_10_(*p*-value)| of methylation and the |log_10_(*p*-value)| of expression (**A**), respectively. Canonical pathways (CP) enriched by hyper-methylated and down-regulated genes in CIMP *vs.* LOW PM cell lines were identified by IPA core analysis (**B**)

Integrative analyses of methylome and transcriptomic data in CIMP *vs.* LOW PM cell lines were performed to study the direct involvement of promoter methylation in the poorly immunogenic profile observed in CIMP PM cell lines (Fig. 6 A). Particularly, an enrichment analysis was conducted on genes (*n* = 217) whose expression was directly down-regulated by hypermethylation (Fig. 6 B) in CIMP *vs.* LOW PM cell lines, identifying inhibited (Z-score ≤ −2) CP mainly associated with class I MHC-mediated antigen processing and presentation, IL-17 signaling, DC maturation and BCR signaling (Additional file 8). Accordingly, the enrichment analysis in CIMP *vs.* LOW PM cells, identified inhibited (Z-score ≤ −2) UR with a predominant influence on tumor necrosis factor-mediated signaling pathway, type II IFN-mediated signaling, regulation of cytokine production involved in inflammatory response, myeloid DC differentiation BP, and on the extracellular matrix organization (Fig. 6 B, Additional file 8).

### Remodeling of methylation profile in CIMP and LOW PM cell lines by DHA treatment

**Fig. 7:**
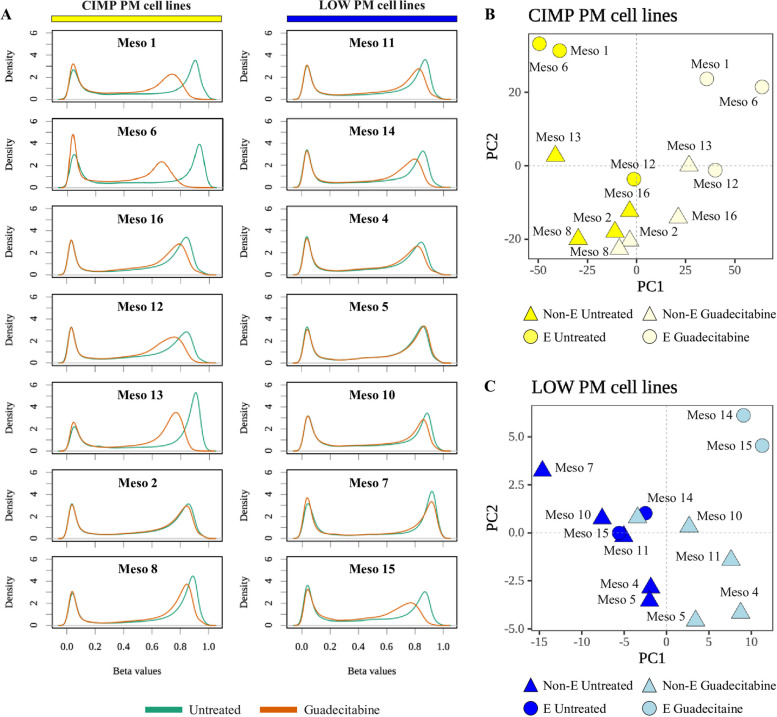
Modulation of methylation in DHA-treated *vs.* untreated PM cell lines. Genomic DNA was extracted from untreated and DHA-treated #14 PM cell lines and used to investigate the methylation profile through Infinium Methylation EPIC array. The density of methylation β value for each CIMP and LOW cell line both at the baseline (green) and after guadecitabine treatment (orange) was plotted (**A**). Dimensionality reduction was performed by applying PCA on the differentially methylated (DM) probes among DHA-treated and untreated CIMP (**B**) and LOW (**C**) PM cell lines. E-PM (circle) or non-E-PM (triangle)

To test whether the hyper-methylated profile of CIMP PM lines could be reverted, we compared the methylation profiles of each CIMP and LOW PM cell line, before and after treatment with guadecitabine. Results demonstrated a more frequent reduction of global methylation in CIMP compared to LOW PM cell lines following DHA treatment (Fig. 7 A); indeed, among the significantly (*p* < 0.05) DM probes, a total of #152,420 and #5,765 probes were hypo-methylated in DHA-treated CIMP and LOW PM cell lines, respectively. PCA stratification analysis revealed that both CIMP and LOW PM cell lines clustered according to DHA treatment, regardless of their histopathological classification (Fig. 7 B, C).

**Fig. 8:**
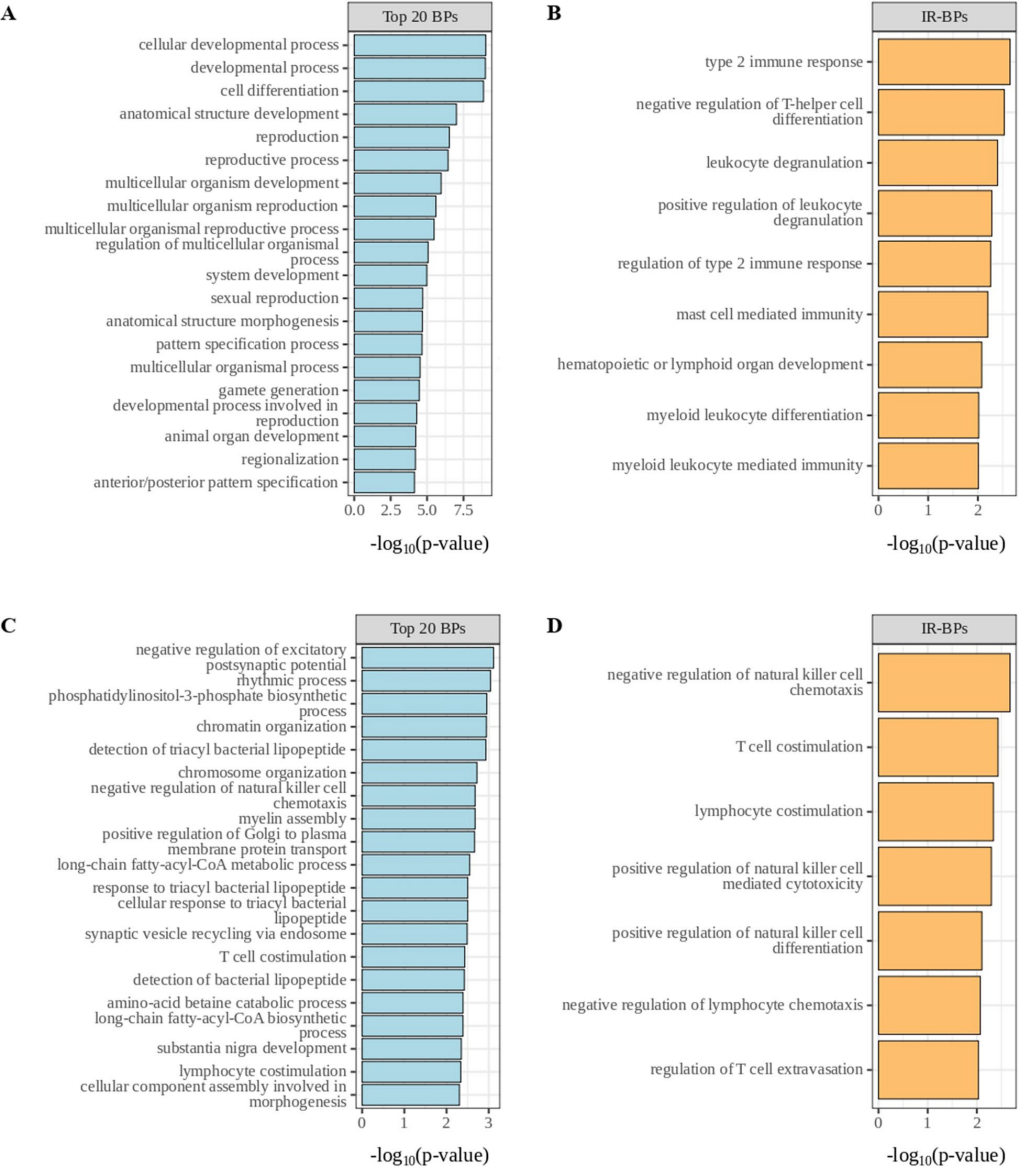
Enrichment analysis of hypo-methylated probes in the promoter region of DHA-treated *vs.* untreated CIMP and LOW PM cell lines. The top 20 biological processes (BP) enriched by hypo-methylated probes of DHA-treated *vs.* untreated genes in CIMP (**A**) and LOW (**C**) PM cell lines. The top 9 immune-related (IR)-BPs enriched by hypo-methylated probes of DHA-treated *vs.* untreated genes in CIMP PM cell lines (**B**), and the top 7 IR-BP enriched by hypo-methylated probes of DHA-treated *vs.* untreated genes in LOW PM cell lines (**D**)

An enrichment analysis was performed on DM probes in DHA-treated *vs.* untreated CIMP and LOW PM cell lines to investigate the BP associated with demethylation. The enrichment analysis of hypo-methylated probes in the promoter region of DHA-treated CIMP PM cells showed an impact on cellular developmental and reproductive/sexual processes as well as multicellular organismal processes (Fig. 8 A), and an enrichment of type 2 immune response, leukocyte degranulation, mast cell mediated immunity and myeloid leukocyte differentiation (Fig. 8 B). The same analysis of hypo-methylated probes of DHA-treated LOW PM cells revealed an impact on chromatin organization, cellular component assembly involved in morphogenesis, lymphocytes co-stimulation and phosphatidylinositol-3-phosphate biosynthetic process (Fig. 8 C), as well as multiple IR-categories, including T cell co-stimulation, regulation of NK cell-mediated cytotoxicity, and regulation of T cell extravasation (Fig. 8 D).

### Transcriptomic profiles of CIMP and LOW PM cell lines after DHA treatment

**Fig. 9:**
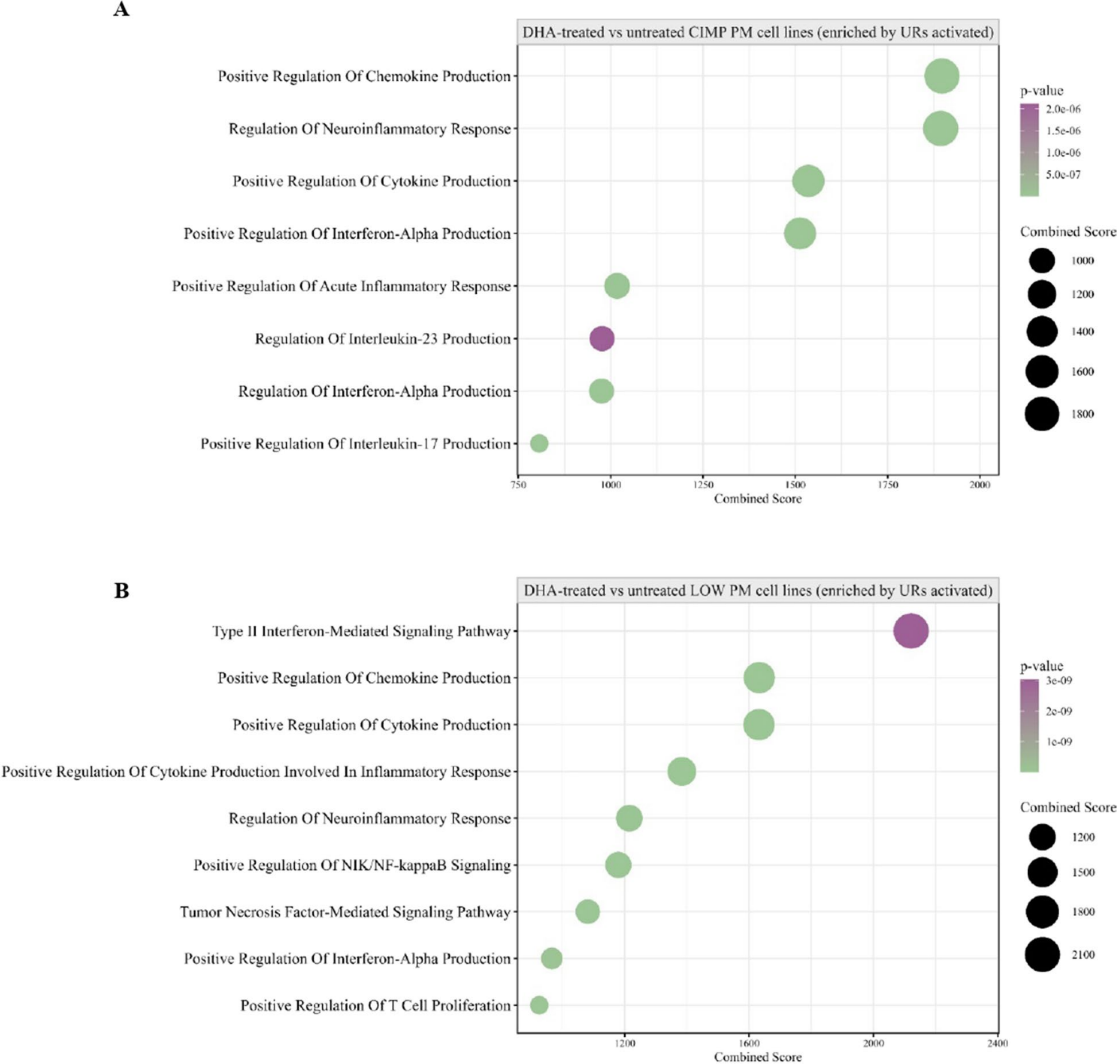
Transcriptomic landscape of CIMP and LOW PM cells after DHA treatment. Dot plots of representative GO terms enriched by activated upstream regulators (UR) in CIMP (**A**) and LOW (**B**) PM cell lines after guadecitabine treatment. The color scale from purple to green indicates the ranges of significant *p*-value (*p* < 0.05), and the size of each single dot correlates with the combined score

**Fig. 10:**
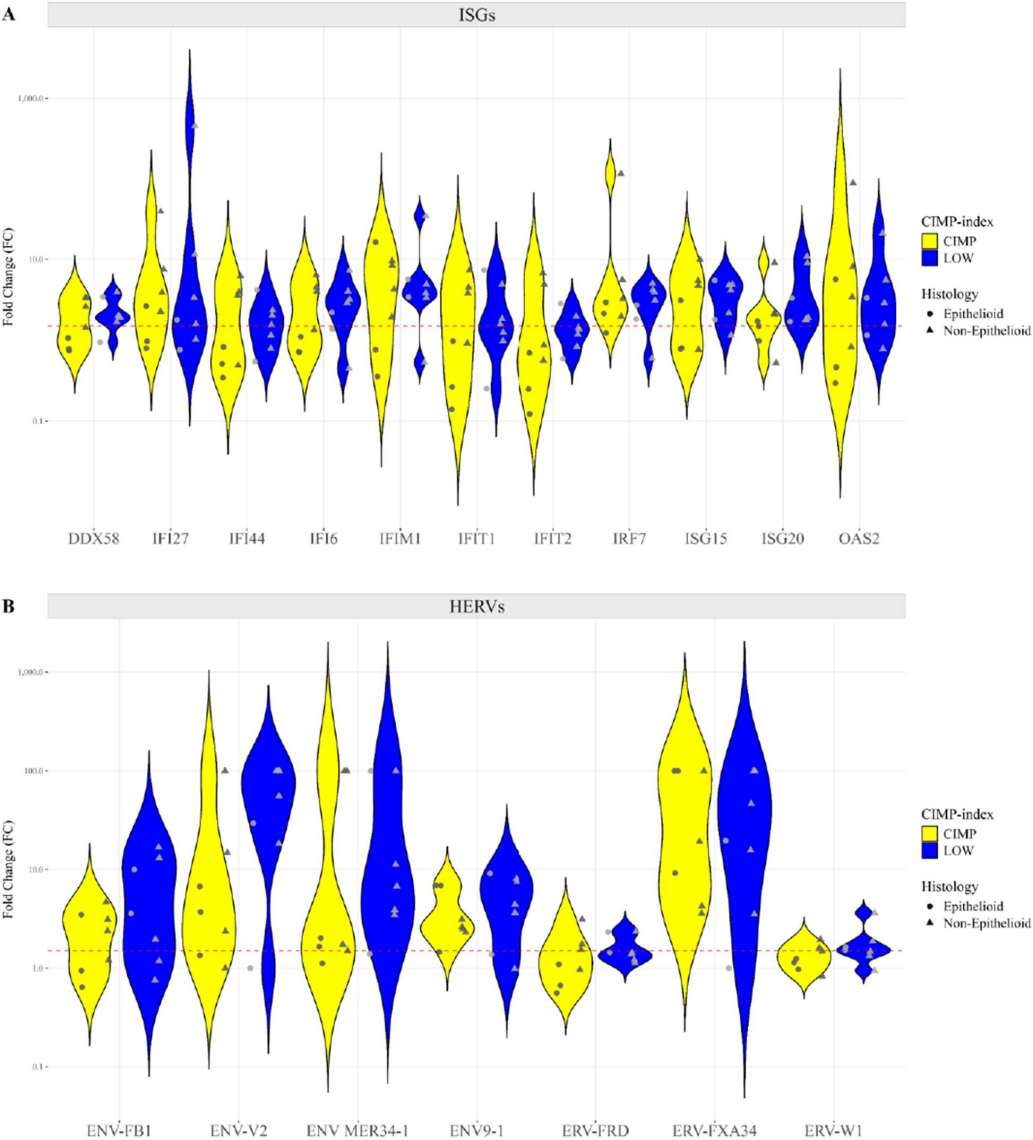
Modulation of viral mimicry-mediating genes expression in DHA-treated *vs.* untreated CIMP and LOW PM cell lines. Starting from retrotranscribed total RNA of untreated and guadecitabine treated PM cell lines (#14), relative Real-Time PCR analyses were performed on ISG (#11) (**A**) and HERV (#7) (**B**) genes. Values are reported in the violin plots as Fold Change (FC) of specific genes in treated *vs.* untreated cells. Each symbol on the graph represents a cell line categorized by its CIMP-index: CIMP (yellow) and LOW (blue), and by its histopathological variant: E (circle) and non-E (triangle). Dashed line (red) represents a FC expression value ≥ 1.5

Transcriptomic analysis was performed to comprehensively explore the effects of guadecitabine on the gene expression profile of CIMP and LOW PM cell lines. Results showed that, among #21,448 investigated genes, #2,358 and #3,034 were significantly (*p* < 0.05) modulated by guadecitabine in CIMP and LOW PM cell lines, respectively. In detail, #1,147 and #1,211 DEGs were up- and down-regulated, respectively, in CIMP PM cell lines, while #1,431 and #1,603 DEGs were up- and down-regulated, respectively, in LOW PM cell lines. It is noteworthy that in the CIMP PM cells, which consistently displayed a constitutive strong immune suppression signature, DHA treatment primarily enhanced the activation of CP related to MHC class I and class II antigen processing and presentation, as well as the cGAS-STING signaling pathway, which is crucial for IFN production and host antiviral responses, beyond the CD28 costimulatory pathway, TCR and NK cell signaling (Additional file 9). Moreover, guadecitabine specifically activated in CIMP PM cells the CDK5 signaling, involved in the inhibition of cell migration [42], and Sphingosine-1-phosphate Signaling that inhibited the growth of mesothelioma cell lines and induced cell cycle arrest at the G0/G1 [43] (Additional file 9). In addition, BP enriched by activated UR were mainly involved in chemokine production, cytokine-mediated signaling and positive regulation of acute inflammatory response (Fig. 9A; Additional file 9). Moreover, only 4 CP were inhibited by guadecitabine in CIMP PM cells, mainly affecting cell invasion and proliferation processes (i.e., Wnt/β-catenin pathway and PPARα signaling) (Additional file 9), and inhibited UR enriched for BP mainly involved in biosynthetic, metabolic processes and in the regulation of TGF-β receptor signaling (Additional file 9). Contextually, DHA treatment also shaped the immune-favorable constitutive profile of LOW PM cell lines. In detail, CP activated by DHA affected a large set of IR-pathways (i.e., TCR signaling, cGAS-STING signaling pathway, DC maturation, MHC class I/II antigen presentation, NK Cell Signaling, ICOS-ICOSL Signaling in T-helper cells) (Additional file 9) and besides these, IFN-mediated signaling pathway and cytokine production were enriched by DHA-activated UR (Fig. 9 B, Additional file 9). Analysis of CP and UR inhibited by DHA, in LOW PM cell lines, were mainly involved in metabolic and proliferative pathways (Additional file 9). One of the main effects exerted by DHA is the induction of the viral mimicry phenomenon through the up regulation of HERV, that activates an antiviral state producing type I and III IFNs and promoting the transcription of ISG [36, 37]. Consistent with the enrichment of BP related to IFN-mediated signaling pathway observed in both DHA-treated CIMP and LOW PM cell lines, a global up-regulation in the expression of HERV (Fig. 10 A) and ISG (Fig. 10 B) genes was observed across all PM cell lines, with a greater extent in LOW PM cell lines. Specifically, although not statistically significant, a higher median fold change (mFC) was observed in treated *vs.* untreated LOW compared to CIMP PM cell lines for RV (mFC = 4.44 *vs.* mFC = 2.39) and for ISG (mFC = 1.97 *vs.* mFC = 1.71), respectively. The modulation of HERV and ISG expression appeared independent of histological classification in both CIMP and LOW PM cell lines (Fig. 10 A, B). Additionally, treatment with DHA positively modulated the protein expression of ISG15 in PM cell lines, inducing or upregulating its expression in two constitutively negative CIMP or positive LOW PM cells, respectively (Additional file 4).

### Integrative analysis of promoter methylation level and transcriptomic profiles in DHA treated vs. untreated CIMP and LOW PM cell lines

**Fig. 11:**
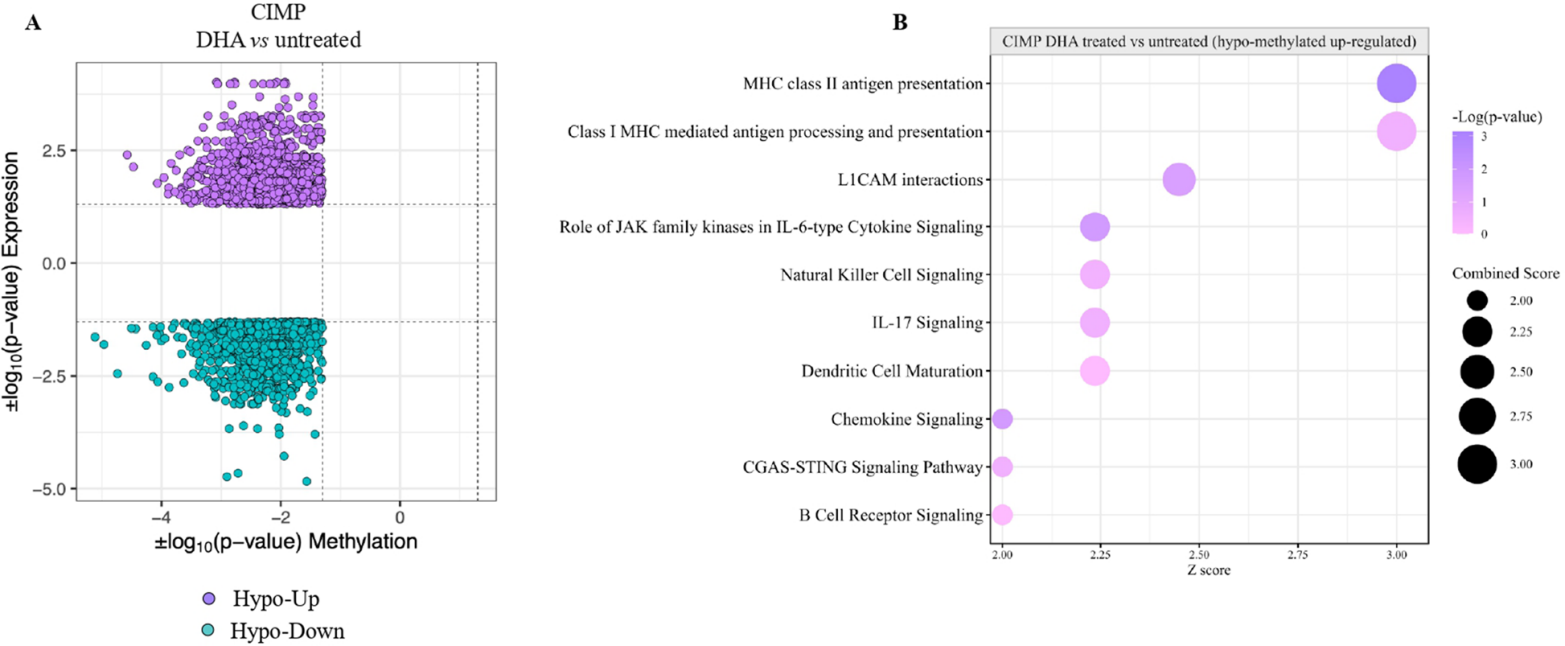
Integrative methylation and expression analysis. The starburst plot shows results of the integrative analysis of promoter methylation and transcriptomic data in DHA treated *vs.* untreated CIMP PM cell lines. x and y axis represent the |log_10_(*p*-value)| of methylation and the |log_10_(*p*-value)| of expression, respectively (**A**). Canonical pathways (CP) enriched by hypo-methylated and u*p-*regulated genes in DHA-treated *vs.* untreated CIMP PM cell lines were identified by IPA analysis (**B**)

To test whether the hyper-methylated immune-silenced profile associated with CIMP PM cell lines was reverted by DHA treatment, we performed integrative analyses of promoter methylation and transcriptomic profiling comparing DHA-treated *vs.* untreated CIMP and LOW PM cell lines. This highlighted genes up regulated by promoter hypomethylation as the most impacted groups (Fig. 11 A). Among the #467 hypo-methylated and up-regulated expressed genes in DHA-treated *vs.* untreated CIMP cells, IPA core analysis revealed that treatment activated (Z-score ≥ 2) CP linked to the immune regulation functions, including the activation of antigen presentation machinery machinery (e.g., MHC class I/II antigen presentation), DC maturation, NK cell signaling, BCR signaling, cGAS-STING signaling pathway (Additional file 10). These results were further supported by the enrichment analysis of the #116 activated (Z-score ≥ 2) UR that identified the activation of immune-related BP along with those involved in the regulation of miRNA transcription, macromolecules metabolic processes and DNA transcription (Additional file 10). Conversely, in LOW PM cell lines, the intersection analysis of promoter methylation and gene expression, identified #49 genes as hypo-methylated and upregulated following DHA treatment. IPA analysis, performed on this small set of genes, revealed enrichment for only one activated UR (CD3-associated response) and four activated CP (i.e., p75 NTR receptor-mediated signaling, co-stimulation by the CD28 family, synaptogenesis signaling pathway, and RHO GTPase cycle).

### Analysis of soluble modulators in DHA-treated CIMP and LOW PM cell lines

**Fig. 12:**
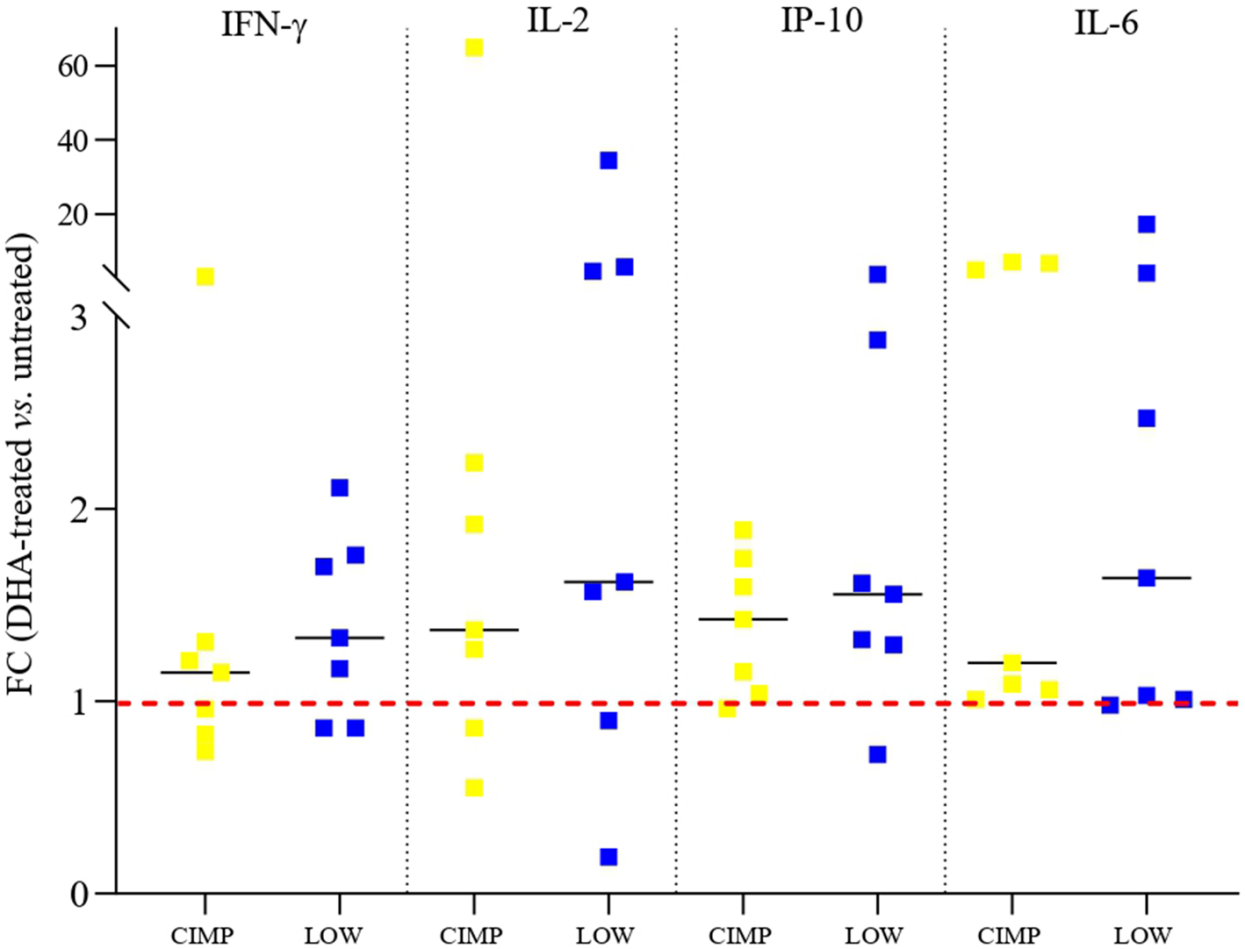
Analysis of soluble modulators in CIMP and LOW PM cell lines. PM cell culture supernatants were collected at the end of guadecitabine treatment from DHA-treated and untreated CIMP and LOW PM cell lines and specific immunoassays were performed to measure levels of soluble immune molecules (i.e., IFN-γ, IL-2, IP-10 and IL-6). Values are reported in the scatter plots as Fold Change (FC) of specific soluble factors in treated vs. untreated cells. Each symbol on the graph represents a cell line categorized as CIMP (yellow) or LOW (blue). Dashed line (red) represents a FC expression value > 1. Statistical analysis was performed by one-way repeated measure ANOVA test

To validate at the protein level the immunomodulatory properties of DHA observed at the transcriptional level in both CIMP and LOW PM cells, the release of soluble immune molecules (i.e., IFN-γ, IL-2, IP-10 and IL-6) was measured in their culture supernatants before and after DHA-treatment by immunoassays. Results showed a global up-regulation (FC > 1) of all investigated modulators, regardless of their methylation class (Fig. 12).

## Discussion

In this study, we utilized a panel of cultured PM cells of different histology to define the contribution of tumor DNA methylation landscape in PM heterogeneity, and to provide preclinical evidence supporting the role of epigenetic remodeling to prospectively improve the efficacy of ICI therapy in PM patients.

To this end, we investigated methylation profiles of PM cell lines to stratify them according to their CIMP-index, in CIMP and LOW classes. Consistent with the notion that the CIMP index is independent from PM morphology [12], our stratification of PM cell lines did not depend upon their E or non-E histological subtype, but rather complements the histopathological classification of PM. Likewise, it was recently demonstrated that the histopathological classification only explains a fraction of the PM heterogeneity, since ploidy, adaptive immune response and CpG island methylation reflected the variations observed in the clinical behavior of PM patients [41].

Moreover, the integrated analysis of epigenetic and transcriptomic characterization of PM cell lines enabled us to explore which biological functions were associated with the two distinct DNA methylation-based classes. This analysis revealed that the CIMP profile included numerous hyper-methylated and silenced genes that impacted BP predominantly involved in shaping a more immune-compromised TME, compared to LOW PM cells. Inhibition of biological processes, such as class I MHC-mediated antigen processing and presentation, type I-II IFN signaling and inflammatory cytokines could, at least in part, explain a different immune escape mechanism associated with methylation classes. Moreover, the different methylation-driven immune pattern identified in CIMP *vs.* LOW could mediate a potential different susceptibility of the two PM classes to ICI therapy, as suggested by their different expression of predictive signatures of ICI response. Reinforcing the potential role of DNA methylation in the shaping tumor methylation profile and its immune contexture, are the results extrapolated from the TCGA database, that confirm the immune-favorable profile of LOW *vs.* CIMP PM lesions. Along this line is the observation that low levels of tumor methylation were strongly associated with the presence of various immune cell subsets, including CD4^+^ regulatory T cells, and lymphocyte infiltration, across different cancer types such as breast cancer, head and neck tumors and lung adenocarcinoma [44]. In line with these findings, Zou et al., demonstrated that the low CD8 + methylation TIL score (enriched CD8 + TILs) predicted better survival in colorectal cancer cohorts [45], reinforcing the concept that the methylation pattern may offer opportunities to model tumor immune compartment. In this context, the dysregulated methylation level can influence the immune function, and increased methylation may act as a biomarker to detect the response level to immunotherapy [46].

Based on these findings and considering the identified association between DNA hypermethylation and the immunosuppressive phenotype of CIMP PM cell lines, we investigated the remodeling activities of guadecitabine on immune-related patterns of CIMP and LOW PM cell lines. Interestingly, treatment with DHA both randomly reverted the methylation-driven immune-compromised profile of CIMP cell lines and reinforced the constitutive immune-favorable profile of LOW PM cell lines. Notably, among the BP commonly affected by DHA treatment in both CIMP and LOW PM cells was the cGAS-STING signaling pathway. This pathway has recently been proposed as a strategy to achieve stronger and more durable efficacy of ICI-based immunotherapy, due to its ability to promote the release of type I IFN and multiple inflammatory cytokines [47]. Another mechanism triggering the IFN-pathway involves the re-expression of HERV sequences, which are transposable elements (TE) covering approximately 8% of the human genome [48]. HERVs play a crucial role in the immune response against tumors, including in the context of ICI therapy [49]. Previous research has demonstrated that DHA can induce HERV re-expression, thereby activating the “viral mimicry” response, which leads to the stimulation of ISGs and antiviral pathways [19, 50]. In line with these findings is the modulation of HERV and ISG genes, considered potent inducers of the IFN-mediated viral mimicry response in PM [51], observed in both CIMP and LOW PM cell lines, following guadecitabine exposure. These findings are consistent with the recent work of Sun and colleagues showing that DHA therapy can unleash viral mimicry in PM, which is further promising since basal activation of this phenomenon is associated with better survival and clinical outcome in PM patients [52]. In addition, guadecitabine enhances the expression of various genes involved in the antigen presentation machinery in both CIMP and LOW PM cell lines. This enhancement is crucial for reinvigorating anti-tumor CD8 T cells, restoring immune control of tumors, and improving immunotherapy efficacy in PM patients [53]. Furthermore, guadecitabine promotes dendritic cell maturation, known for their potent antigen-presenting capacity [54], and NK cell signaling, which is being studied for its anti-tumor activity as an immunotherapeutic approach for PM. Beyond the reactivation of most IR-functions, influenced by hypomethylation of specific gene promoter regions, it is fair to mention the inhibitory action of guadecitabine on the pro-metastatic WNT/β-catenin signaling. This pathway is deeply implicated in PM pathogenesis [55] and correlated with immune exclusion in several human cancers [56]. Additionally, guadecitabine negatively affected the major downstream effectors of the Hippo pathway, on the YAP/TAZ-axis, known to confer a proliferation advantage on PM cells via transcriptional regulation of cell cycle-related genes, as well as on tumorigenesis, progression, metastasis, and recurrence (57). Comprehensively, as already observed in previous *vitro* studies [16], immune modulation emerged as the preponderant beneficial effect of guadecitabine in PM cell lines also considering the new PM classification based on the extent of tumor DNA methylation identified in this study, that is independent from the PM histological subtype. This novel methylation-based classification has the potential to redefine how PM should be stratified and treated by providing insights into epigenetic markers that enable the identification of distinct subgroups with differentiated responses to anti-tumor treatments, including ICIs and other therapies. Furthermore, the association between the tumor methylome and immune activity in the microenvironment across multiple cancer types supports the extension of this methylation-based stratification to drive personalized treatment decisions in cancers of various histotypes, particularly those with limited effective therapies.

## Conclusions

The novelty of our present study demonstrates that tumor methylome classification, associated with the immune phenotype of PM cells, could serve as a better molecular predictor of clinical outcome to ICI therapy of PM patients, regardless of their tumor histotype. This perspective has the potential to be translated into clinical practice, optimizing the selection of PM patients that could benefit more from ICI therapy also independently from the tumor histotype. Finally, although further confirmatory analyses are granted in the clinical setting of PM, the pharmacologic immune remodeling induced by DHA in both CIMP and LOW PM methylation classes could improve the efficacy of ICI in PM patients, laying the foundation for the use of DHA in prospective clinical trials of epigenetic-based ICI therapy.

## Supplementary Information

Additional file 1.

Additional file 2: Fig. 1. Distribution of PM cell lines based on DM probes. Dimensionality reduction was performed applying PCA on the DM probes among all PM cell lines. Each symbol on the graph represents a cell line categorized by its methylation status: CIMP (yellow) and LOW (blue) and by its histopathological variant: E-PM (circle) and non-E-PM (triangle).

Additional file 3: Fig. 2. Distribution of DNA methylation level in relation to CpG island regions and genomic regions in CIMP vs. LOW PM cell lines. Bar graphs represented the distribution of hyper-methylated (A, C) or hypo-methylated (B, D) probes, identified in CIMP vs. LOW PM cell lines, across different CpG island regions (A, B) and functional regions including first exon (1stExon), 3’ untranslated region (3’UTR), 5’ untranslated region (5’ UTR), gene body (Body), exon boundaries (ExonBnd), 1500 bases upstream of the transcription site (TSS1500) and 200 bases upstream of the transcription site (TSS200) (C, D).

Additional file 4: Fig. 3. Protein expression of ISG15 in untreated and DHA-treated CIMP and LOW PM cell lines. Protein lysates isolated from the 4 investigated PM cell lines (CIMP: Meso 6 and Meso 8; LOW: Meso 5 and Meso 15) were run on SDS-PAGE under reducing conditions and blotted onto polyvinylidene fluoride membranes. Membranes were then incubated with anti-ISG15 specific antibody and further processed to be developed by the enhanced chemiluminescence technique.

Additional file 5.

Additional file 6.

Additional file 7: Fig. 4. Immune phenotypic profiles in CIMP and LOW TCGA-MESO cohort. Tumor microenvironment deconvolution of immune and stromal cell fractions was performed for CIMP (yellow) and LOW (blue) PM tissues from the TCGA-MESO cohort, considering the top 25% hyper-methylated CIMP lesions and the top 25% hypo-methylated LOW PM lesions.

Additional file 8.

Additional file 9.

Additional file 10.

## Data Availability

No datasets were generated or analysed during the current study.

## Abbreviations

BCR: B cell receptor
BP: Biological processes
cGAS-STING: Cyclic GMP-AMP synthase (cGAS)-stimulator of interferon genes (STING)
CIMP: CpG island methylator phenotype
CP: Canonical pathways
CTLA-4: Cytotoxic T lymphocyte antigen-4
DC: Dendritic cell
DHA: DNA hypomethylating agent
DM: Differentially methylated
E: Epithelioid
E-score: Epithelioid score
HERV: Human endogenous retrovirus
ICI: Immune-checkpoint inhibitors
ICOS: Inducible Co-Stimulator
IPA: Ingenuity pathway analysis
IR: Immune-related
ISG: Interferon stimulating gene
mAb: Monoclonal antibody
MDSC: Myeloid derived suppressor cells
MHC: Major histocompatibility complex
NES: Normalized enrichment scores
NK: Natural killer
non-E: Non-epithelioid
OS: Overall survival
PCA: Principal component analysis
PD-1: Programmed cell death
PM: Pleural mesothelioma
S-score: Sarcomatoid-score
TCR: T cell receptor
TE: Transposable element
TGF-β: Transforming growth factor beta
Th17: T-helper (Th)-17
Th2: T-helper (Th)-2
TIL: Tumor-infiltrating lymphocyte
TLS: Tertiary lymphoid structure
TME: Tumour microenvironment
UR: Upstream regulators
