# Supplementary figures and images for: DNA methylation status classifies pleural mesothelioma cells according to their immune profile: implication for precision epigenetic therapy
Source: J Exp Clin Cancer Res. 2025 Feb 18;44:58. doi: 10.1186/s13046-025-03310-0 (PMC11834541; doi:10.1186/s13046-025-03310-0)

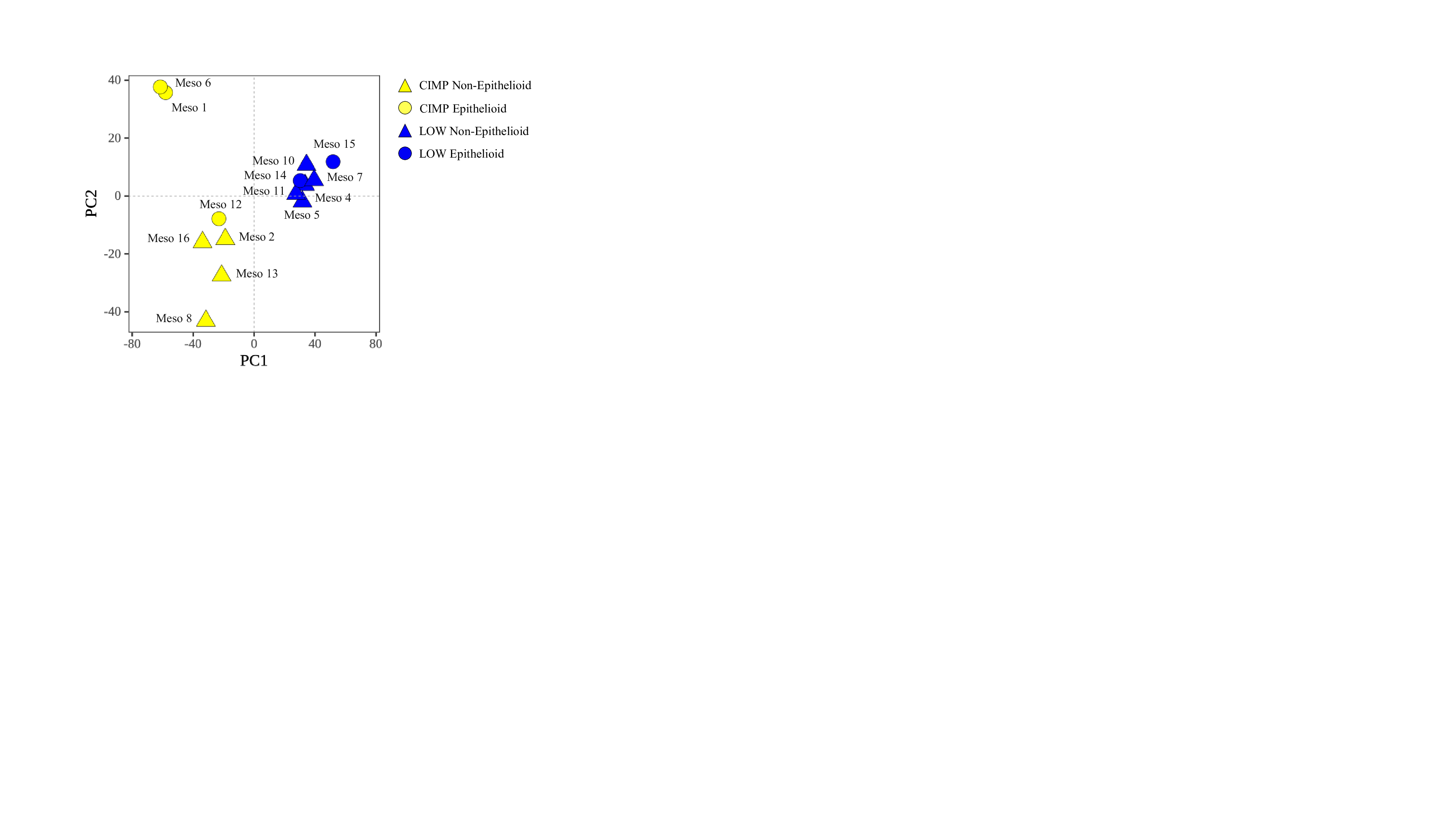

Supplement: Supplementary file 2 — Additional file 2: Fig. 1. Distribution of PM cell lines based on DM probes. Dimensionality reduction was performed applying PCA on the DM probes among all PM cell lines. Each symbol on the graph represents a cell line categorized by its methylation status: CIMP (yellow) and LOW (blue) and by its histopathological variant: E-PM (circle) and non-E-PM (triangle). [file 13046_2025_3310_MOESM2_ESM.jpg]

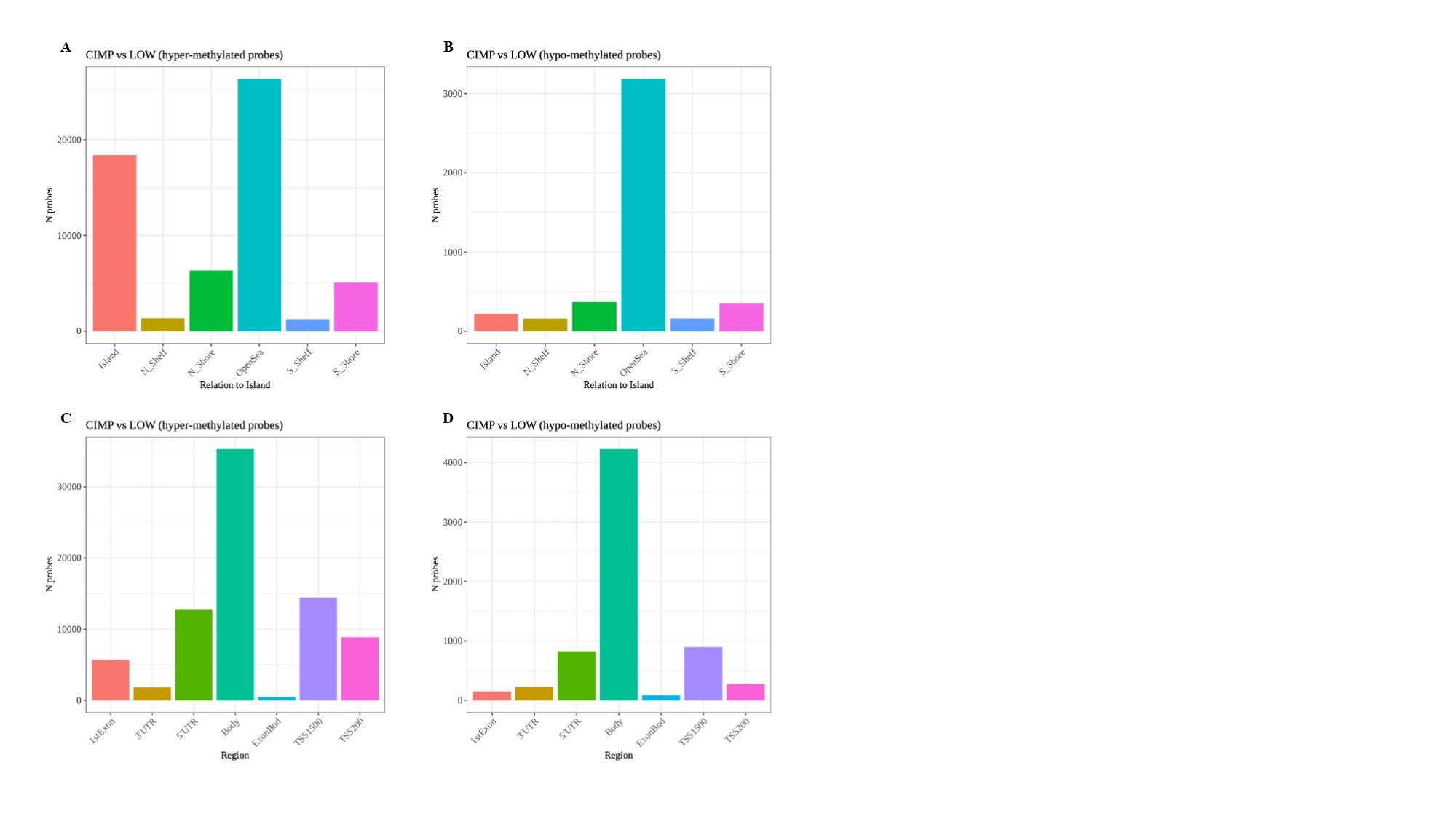

Supplement: Supplementary file 3 — Additional file 3: Fig. 2. Distribution of DNA methylation level in relation to CpG island regions and genomic regions in CIMP vs. LOW PM cell lines. Bar graphs represented the distribution of hyper-methylated (A, C) or hypo-methylated (B, D) probes, identified in CIMP vs. LOW PM cell lines, across different CpG island regions (A, B) and functional regions including first exon (1stExon), 3’ untranslated region (3’UTR), 5’ untranslated region (5’ UTR), gene body (Body), exon boundaries (ExonBnd), 1500 bases upstream of the transcription site (TSS1500) and 200 bases upstream of the transcription site (TSS200) (C, D). [file 13046_2025_3310_MOESM3_ESM.jpg]

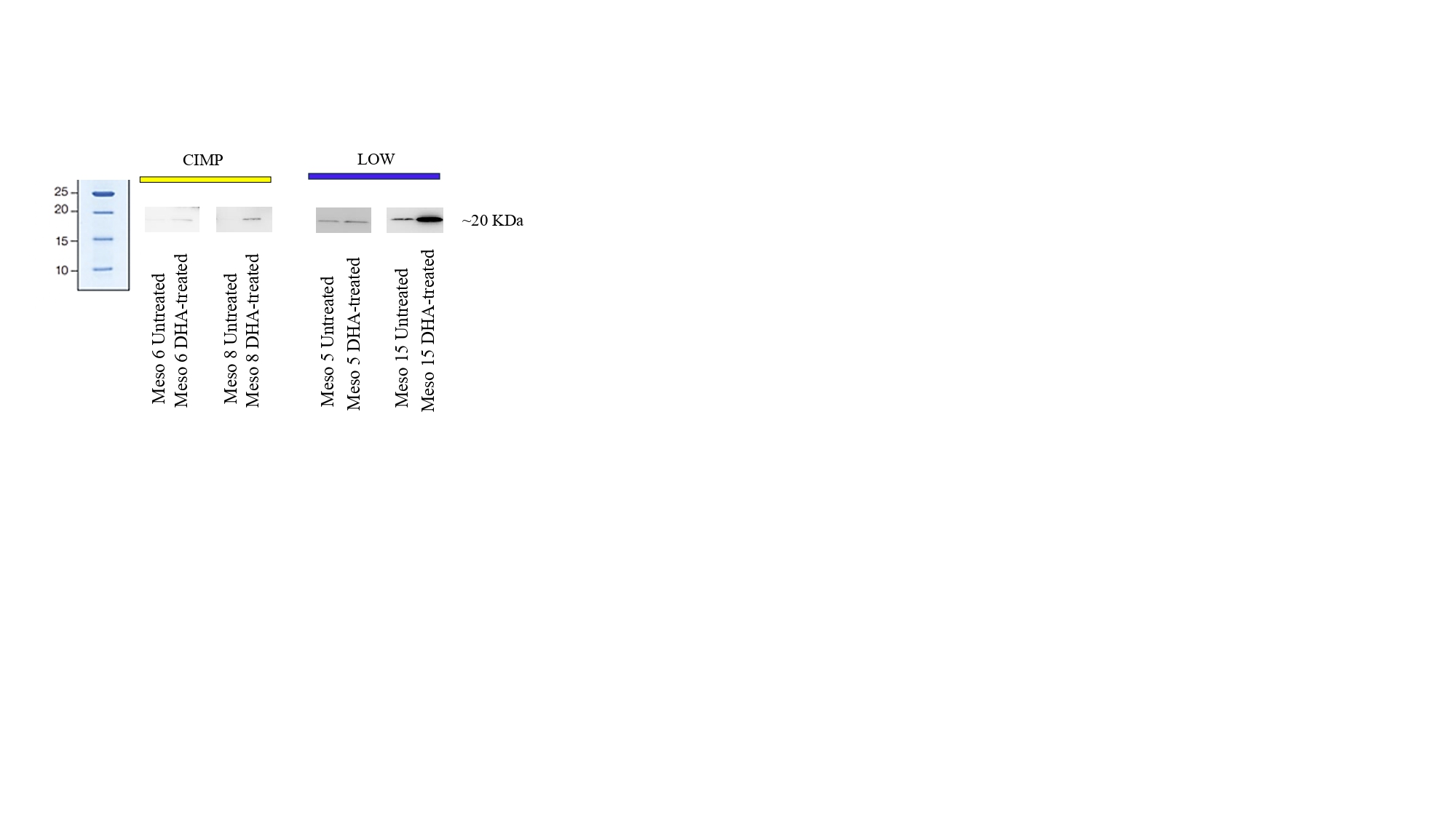

Supplement: Supplementary file 4 — Additional file 4: Fig. 3. Protein expression of ISG15 in untreated and DHA-treated CIMP and LOW PM cell lines. Protein lysates isolated from the 4 investigated PM cell lines (CIMP: Meso 6 and Meso 8; LOW: Meso 5 and Meso 15) were run on SDS-PAGE under reducing conditions and blotted onto polyvinylidene fluoride membranes. Membranes were then incubated with anti-ISG15 specific antibody and further processed to be developed by the enhanced chemiluminescence technique. [file 13046_2025_3310_MOESM4_ESM.jpg]

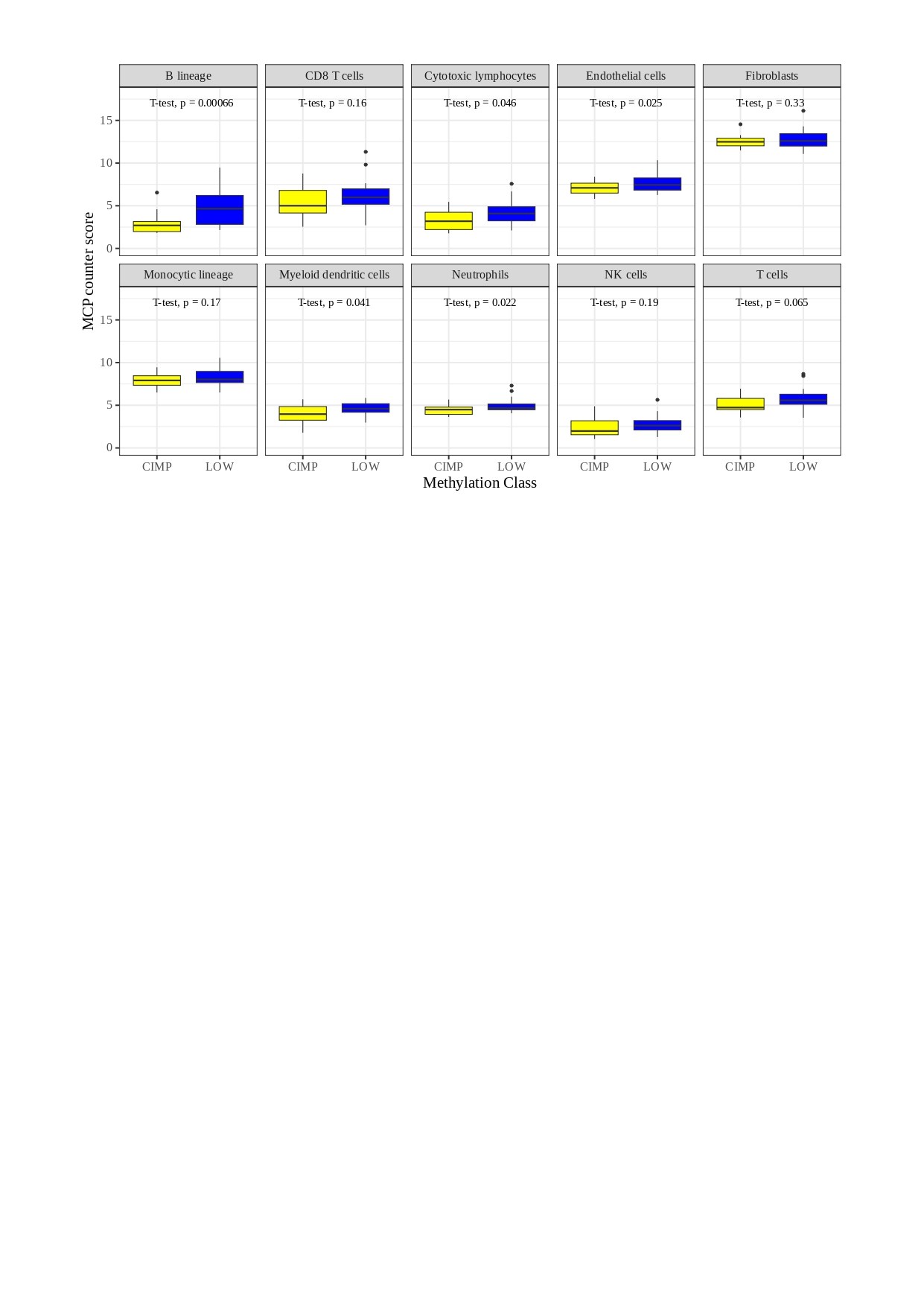

Supplement: Supplementary file 7 — Additional file 7: Fig. 4. Immune phenotypic profiles in CIMP and LOW TCGA-MESO cohort. Tumor microenvironment deconvolution of immune and stromal cell fractions was performed for CIMP (yellow) and LOW (blue) PM tissues from the TCGA-MESO cohort, considering the top 25% hyper-methylated CIMP lesions and the top 25% hypo-methylated LOW PM lesions. [file 13046_2025_3310_MOESM7_ESM.jpg]
